# A pontomesencephalic-mesolimbic circuit modulates activity-based anorexia

**DOI:** 10.1126/sciadv.aee1476

**Published:** 2026-09-23

**Authors:** Beibei Peng, Xu Gao, Yan Chen, Yushi Xin, Yuxiao Zhang, Kexin Yu, Mingli Lu, Wenyan Li, Deqi Yang, Chaofei Bao, Shuai Liu

**Affiliations:** ^1^ Shanghai Changning Mental Health Center, Affiliated Mental Health Center, East China Normal University, Shanghai 200335, China.; ^2^ NYU-ECNU Institute of Brain and Cognitive Science at NYU Shanghai, Shanghai 200062, China.; ^3^ Shanghai Key Laboratory of Brain Functional Genomics (Ministry of Education), School of Psychology and Cognitive Science, East China Normal University, Shanghai 200062, China.

## Abstract

Anorexia nervosa (AN) is a psychiatric disorder with a high mortality rate. The compulsive nature of the disorder leads to an emerging dopamine (DA)–centered hypothesis. However, the circuit mechanism of AN remains elusive. By examining a pontomesencephalic-mesolimbic circuit, which is implicated in anorexia-like and reinforcement behaviors in naïve mice, we found that this pathway modulates activity-based anorexia (ABA), a well-established animal model of AN. Specifically, glutamatergic lateral parabrachial nucleus (LPBN) neurons indirectly inhibited medial ventral tegmental area (VTA) DA neurons through local GABAergic interneurons. Chronic activation of the LPBN-VTA circuit exacerbated ABA symptoms, whereas circuit suppression alleviated them, demonstrating its sufficiency and necessity. Moreover, VTA DA neurons exhibited impaired high-conductance calcium- and voltage-dependent potassium (BK) channel currents. Pharmacological enhancement of this channel improved the retention rate in ABA mice. Our results elucidate the critical role of the LPBN-VTA circuit and potential channel pathology, which may serve as a key to the development of drug treatments and intervention strategies for AN.

## INTRODUCTION

Anorexia nervosa (AN) is a psychiatric disorder with a high mortality rate. The core symptoms of the AN are characterized by self-starvation and are often accompanied by excessive exercise (*1*–*3*). AN is associated with various physical and mental issues, placing a huge burden on patients (*4*). There is now no evidence-based pharmacological treatment available for AN due to the unclear neuropathological mechanism and the lack of a therapeutic target (*1*, *5*). Therefore, understanding the neurophysiological basis of AN is key to developing drug treatments and intervention strategies.

The dopaminergic system has been implicated in the development and maintenance of compulsive behaviors (*6*, *7*). The compulsivity of AN has led to an emerging dopamine-centered hypothesis, with a growing body of literature showing the involvement of the dopaminergic system in the onset, progression, and response to treatment of AN, in aspects ranging from behavioral patterns to neuroimaging signatures (*8*–*14*). However, the key dopamine neural circuit mechanism underlying AN remains elusive. To address the problem, we propose that the key neural circuit responsible for AN should satisfy the following two criteria: (i) activation of the neural circuit inhibits food intake; and (ii) activation of the neural circuit induces behavioral reinforcement. Although it is rare to have such a counter-evolutionary disruption, external factors combined with a susceptible genetic background can trigger the circuit to be the key neural substrate for AN (*1*, *5*, *15*).

One potential circuit from the lateral parabrachial nucleus (LPBN) to the ventral tegmental area (VTA) emerges from previous studies. First, the LPBN receives energy homeostatic information from the hypothalamus, a critical hub that senses and relays the circulating orexigenic and anorexigenic signals (*16*). The LPBN encodes food neophobia (*17*, *18*) and positive valence in the hungry state (*19*). Second, the LPBN neurons project to the VTA, a dopaminergic region critical for reward processing and behavioral reinforcement (*20*). Third, dysfunction of the mesolimbic dopamine system has been implicated in both patients with AN and animal models (*12*, *21*–*26*). In the present study, we therefore systematically characterize the functional architecture of the LPBN-VTA-NAc circuit based on the criteria proposed above and test its causal contribution to AN phenotypes in a translationally relevant ABA model using in vivo and in vitro electrophysiology, optogenetics, chemogenetics, and fiber photometry.

## RESULTS

### LPBN-VTA circuit bidirectionally modulates food intake in fasted mice

The LPBN is an essential nucleus for modulating food intake behaviors (*27*). Lesions in LPBN neurons induced hyperphagia and hyposensitivity to the anorectic peptides (*28*, *29*), indicating their necessary roles in relaying satiety information. Given its abundant innervations to the limbic pathway (*20*), the appetite suppressing information can be transmitted to the VTA, a critical area for reward and motivation, and further reinforced in the mesolimbic system.

**Fig. 1.**
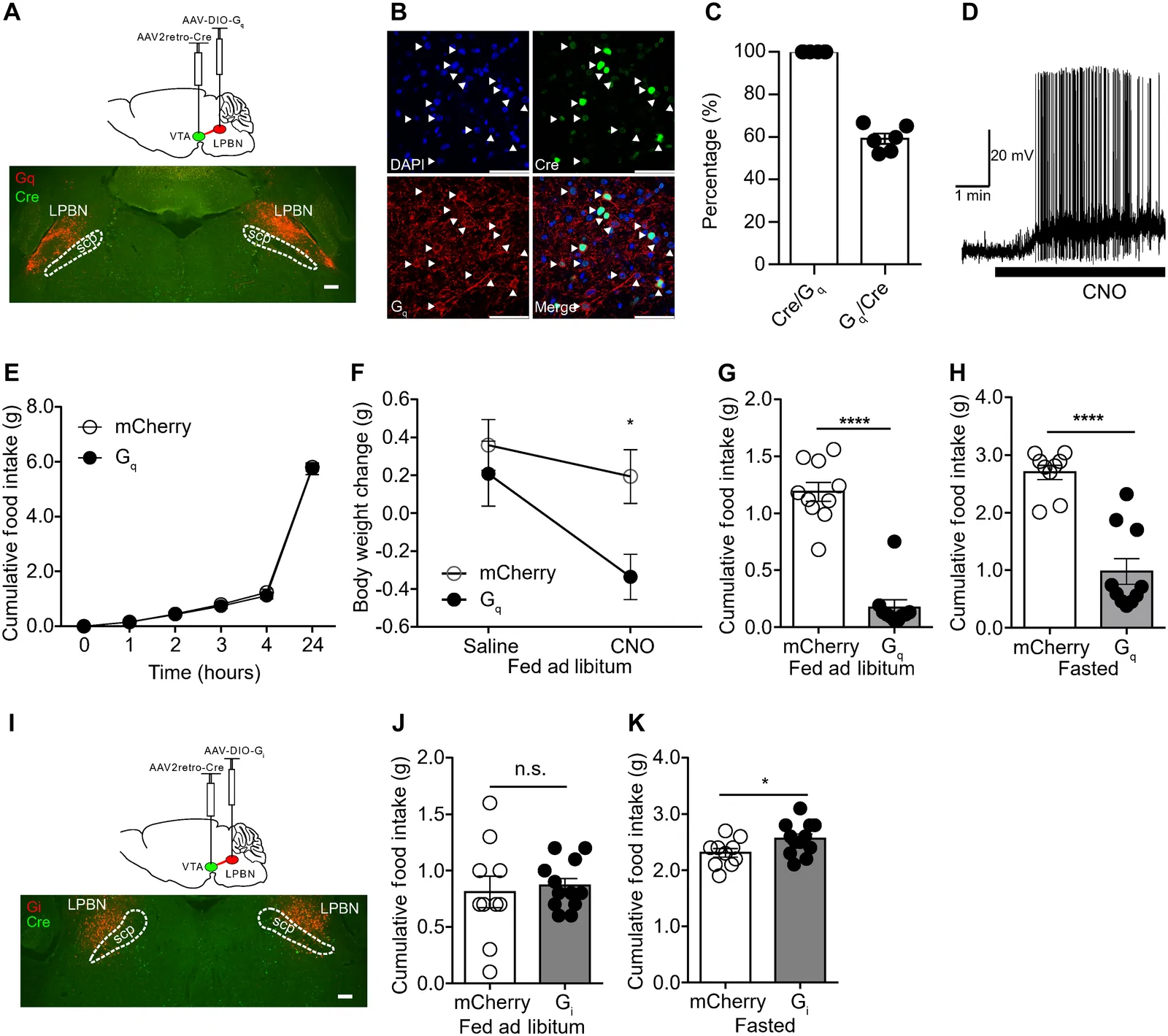
The LPBN-VTA circuit bidirectionally modulates food intake in fasted mice. (**A**) Schematic illustration shows the strategy to target the LPBN-VTA circuit by G_q_-expression virus (top) and the expression of G_q_ was limited in the LPBN (bottom). Scale bar, 100 μm. (**B**) Retrograde Cre signal (green) and Cre-dependent G_q_ signal (red) were colocalized in LPBN at high magnification image. Scale bars, 50 μm. DAPI, 4′,6-diamidino-2-phenylindole. (**C**) Quantification of overlap rate between Cre and G_q_ in LPBN. All G_q_-expressing neurons expressed Cre. Cre-expressing neurons (59%) colocalized with G_q_. Data from six mice. (**D**) Example trace showing that CNO (10 μM) increased the firing rate of G_q_-positive neurons. (**E**) Systemic administration of saline did not change food intake in G_q_ mice. (**F**) Activation of the LPBN-VTA circuit decreased body weight one day after CNO injection. (**G**) Activation of the LPBN-VTA circuit decreased 4-hour cumulative food intake after CNO injection in G_q_ ad libitum fed mice. (**H**) Activation of the LPBN-VTA circuit decreased 4-hour cumulative food intake after CNO injection in G_q_ fasted mice. (**I**) Schematic illustration shows the strategy to target the LPBN-VTA circuit by Gi-expressing virus (top) and its expression patterns in LPBN (bottom). Scale bar, 100 μm. (**J**) Inhibition of the LPBN-VTA circuit did not alter 3-hour cumulative food intake in ad libitum fed mice. (**K**) Inhibition of the LPBN-VTA circuit increased 4-hour cumulative food intake after CNO injection in fasted mice.

To test whether the LPBN-VTA circuit meets the first criterion proposed in our hypothesis, we first manipulated it using G_q_-coupled designer receptors exclusively activated by designer drugs (DREADDs) virus to observe how it affects food intake (Fig. 1A). Cre-dependent G_q_ virus was exclusively expressed in the LPBN (Fig. 1A). Quantification of the coexpression rate between retrograde Cre signal from VTA and Cre-dependent G_q_ signal in LPBN demonstrated that all G_q_-expressing neurons in LPBN coexpressed with Cre, suggesting no leak expression of G_q_ virus (Fig. 1, B and C). G_q_ fibers from LPBN were observed in the VTA (fig. S1A), confirming that the retrograde virus was correctly injected as well as the existence of the LPBN-VTA pathway. A previous study has demonstrated that abundant peri–locus coeruleus (peri-LC) neurons project to VTA and play roles in consumption behaviors (*30*). Consistent with this observation, we also found many VTA-projecting neurons located in the peri-LC. However, no G_q_ signal was observed in the peri-LC, indicating the peri-LC–VTA was not involved in our studies (fig. S1B). Exogenous administration of CNO to LPBN containing brain slices increased the firing rates of VTA projecting LPBN neurons (Fig. 1D). No constitutive activity (*31*) of G_q_ was observed following the intraperitoneal injection of saline during the cumulative food intake test (Fig. 1E). Activation of the LPBN-VTA circuit decreased body weight one day after CNO administration (Fig. 1F). Intriguingly, we found that CNO administration decreased cumulative food intake in both ad libitum fed (Fig. 1G) and fasted G_q_-expressing mice (Fig. 1H), suggesting that the LPBN-VTA circuit plays a role in appetite suppressing behaviors. To test the essential role of the LPBN-VTA circuit in appetite suppressing behaviors, we suppressed this circuit using a G_i_ virus (Fig. 1I). We found that inhibition of this circuit did not change cumulative food intake in ad libitum mice (Fig. 1J); however, a significant increase in cumulative food intake was observed in fasted mice (Fig. 1K). The above results indicate that the LPBN-VTA circuit can bidirectionally modulate the food intake in fasted mice. Calcitonin gene-related peptide (CGRP)–expressing neurons are abundant in the outer external lateral subdivision of the PBN and are pivotal for the suppression of food intake (*32*). Consistent with the previously finding (*33*), we did not observe the coexpression of CGRP and retrograde Cre in the LPBN in our study (fig. S1C), suggesting that VTA-projecting neurons are not CGRP-expressing neurons.

### Transient activation of LPBN-VTA terminals is reinforcing

**Fig. 2.**
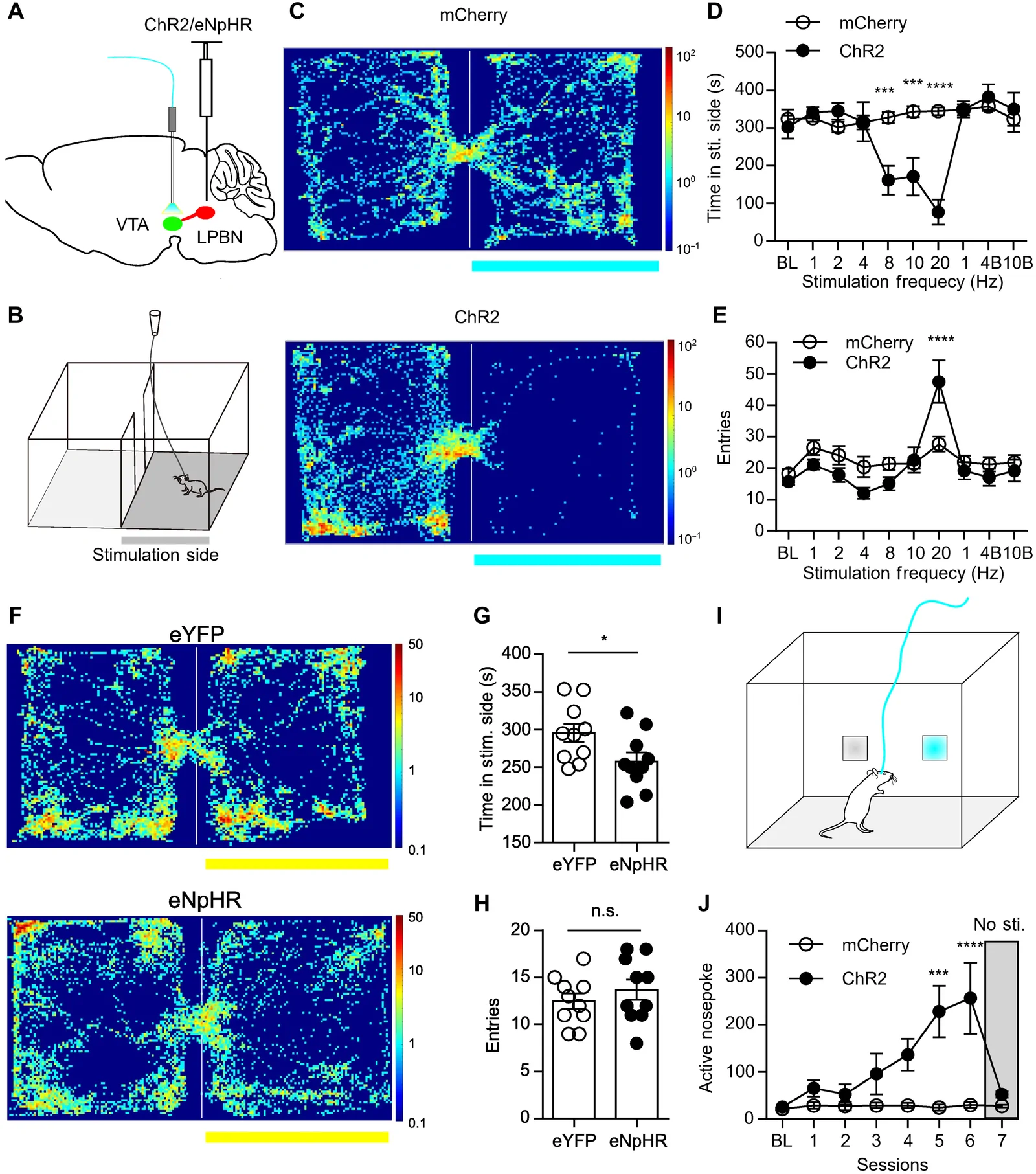
The transient activation of the LPBN-VTA circuit is reinforcing. (**A**) Schematic illustration of circuit targeting using optogenetics. ChR2/eNpHR or its control mCherry/eYFP AAV was injected into the LPBN, and optical fiber was bilaterally implanted into the VTA to activate or inhibit the circuit, respectively. (**B**) Schematic of the real-time place preference (RTPP) test apparatus. (**C**) Example heatmap of mice exploring the apparatus with 20-Hz blue laser (473 nm) activation. ChR2 mice (bottom) showed less time in the blue laser paired side (blue bar) compared with the mCherry mice (top). However, mice spent more time in the area near the entrance to the paired side indicating more attempts to enter the paired side. The values on the right side of the graph are the frame numbers of the movie (sampling rate, 30 Hz). (**D**) Mice spent less time on the blue laser paired side when the LPBN-VTA circuit was activated in various frequency stimulation patterns. (**E**) Mice increased entries into the blue laser paired side when the circuit was stimulated at high frequency (20 Hz). (**F**) Example heatmap of mice exploring the RTPP apparatus with yellow laser (589 nm) inhibition. eNpHR mice decreased exploration time without affecting the entries to the yellow light paired side compared with the eYFP mice. The yellow bar beneath each figure represents the side paired with yellow light. (**G**) Inhibition of the LPBN-VTA circuit decreased exploration time in the yellow laser paired side. (**H**) Inhibition of LPBN-VTA had no effect on entries to the paired side. (**I**) Schematic of the optogenetic self-stimulation test. The active screen nose poking triggers a transient 20-Hz blue laser stimulation, which activates the LPBN-VTA circuit. (**J**) ChR2 mice showed more nose-poke responses in the optogenetic self-stimulation test over consecutive training sessions

To test whether the LPBN-VTA circuit meets the second criterion proposed in our hypothesis, we then examined whether the circuit induces a contextual reinforcement using real-time place preference (RTPP) for optical self-stimulation as previously described (*34*). Briefly, ChR2/eNpHR or their control mCherry/eYFP virus was injected into the LPBN. Optical fibers were then bilaterally implanted into the VTA to activate/inhibit the circuit, respectively (Fig. 2A). During the RTPP test, the mice were allowed to freely explore the two chambers of the apparatus. Upon entering the stimulation chamber, preprogrammed laser at 473-nm (for ChR2) or 589-nm wavelength (for eNpHR) was delivered simultaneously through the fiber into the VTA (Fig. 2B). Intriguingly, activation of this circuit at frequencies from 8 to 20 Hz decreased the exploratory time in the light-paired compartment (Fig. 2, C and D). Counterintuitively, mice increased their entries into the light-paired compartment when light stimulation was delivered at 20 Hz (Fig. 2, C and E, and movie S1). Inhibition of this circuit using eNpHR in the RTPP test decreased the time spent exploring the light-paired compartment without affecting entry behaviors (Fig. 2, F to H). The increased number of entries and reduced exploratory time spent in the light-paired compartment during 20-Hz stimulation in ChR2-expressing mice led us to investigate whether these mice are more involved in transient activation of this circuit rather than continuous stimulation (*35*, *36*). We thus used an optical intracranial self-stimulation (ICSS) paradigm in an operant chamber. Mice received a brief light stimulation (two pulses at 20 Hz) when nose poking the active touchscreen in the chamber (Fig. 2I). Mice significantly increased their nose-poking response to the active touchscreen when paired with photostimulation, supporting our hypothesis that mice are involved more transient activation of the LPBN-VTA (Fig. 2J and movie S2). Similar results were observed in female adolescent Balb/c mice, suggesting that the reinforcing property of the LPBN-VTA circuit transient activation is not specific to sex, age, or strain (fig. S1, D and E). Notably, the number of nose pokes returned to baseline on the following day when photostimulation was terminated (Fig. 2J), indicating that these stimulations had no long-lasting effect. We thus used the conditioned paired preference/aversion test (CPP/CPA) to verify the long-lasting effects of LPBN-VTA activation (fig. S1F). After repeatedly pairing the context cue with the optical activation of this circuit, we did not observe any place preference or aversion in ChR2 mice in the test (fig. S1G). This may be because CPP/CPA involves a delay between the reinforcing stimulus and the test, relying on complex and widespread neural networks for memory and association, in addition to the LPBN-VTA circuit activation (*37*). Overall, the mice instrumentally responded to transient stimulation of the LPBN-VTA pathway, suggesting that this stimulation is reinforcing.

Together, the two sets of results above support the LPBN-VTA circuit as a key pathway in AN by meeting the two criteria outlined in our proposed hypothesis: activation of the pathway suppresses food intake and transient stimulation produces behavioral reinforcement. Based on this, we next investigated the role of this circuit in the pathogenesis of anorexia using the ABA mouse model.

### The LPBN-VTA circuit mediates the food intake and retention rate in the ABA model

**Fig. 3.**
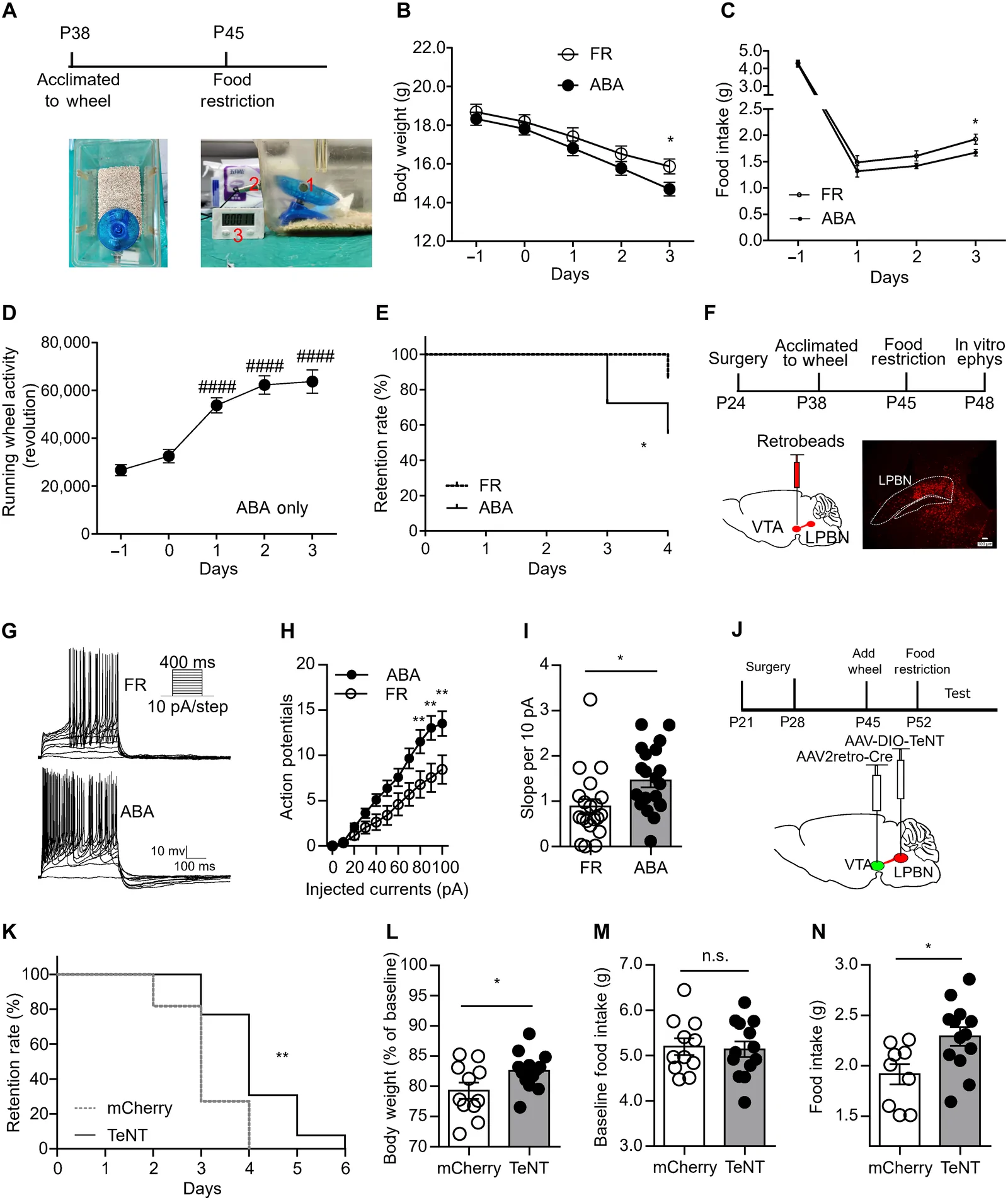
Inhibition of the overactivated LPBN-VTA circuit improves food intake and the retention rate in the ABA model. (**A**) The timeline and equipment setup for the ABA model. Adolescent mice were given unlimited access to running wheel (1 on the bottom right, unlocked for ABA and locked for FR) during the food restriction. Bottom left is the top view of the wheel. The running wheel activity was recorded through a magnetic sensor (2 on the bottom right) and displayed on a digital monitor (3 on the bottom right). (**B**) Body weight changes in ABA and FR mice. (**C**) Food intake changes in ABA and FR mice. (**D**) Running wheel activity during the food restriction, mice increased running wheel activity after food restriction. ####*P* < 0.0001 represents the significant difference with baseline. (**E**) Retention rates in the ABA and FR mice. (**F**) Timeline of ABA in vitro electrophysiology (ephys). Retrobeads were injected into the VTA (bottom left) to target the VTA-projecting LPBN neurons (bottom right). Scale bar, 100 μm. The excitability of the VTA-projecting LPBN neurons was tested by recording LPBN-containing brain slices obtained from ABA or FR mice. (**G**) Representative excitability traces of VTA-projecting LPBN neurons in the ABA and FR mice. (**H**) Excitability of VTA-projecting LPBN neurons in the ABA and FR mice. (**I**) The slope of the number of spikes per 10 pA. (**J**) Timeline of silence the LPBN-VTA circuit in ABA mice using TeNT. (**K**) Retention rate improved by the TeNT silence of the LPBN-VTA circuit in ABA mice. (**L**) Silencing the LPBN-VTA pathway alleviated weight loss in ABA mice during restriction period. (**M**) Silencing LPBN-VTA did not change baseline food intake. (**N**) Food intake changed by silence of the LPBN-VTA circuit during food restriction period.

ABA is a widely used animal model to investigate AN (*38*). Because the LPBN-VTA circuit is involved in appetite suppression and reinforcing behaviors, we investigated its role in ABA mice. We hypothesize that the basal activity of this circuit is enhanced in ABA mice, which could strengthen their appetite-suppressing behavior while also providing reinforcing properties. This is supported by clinical observations that patients with AN perceive self-starvation as a reinforcer (*39*, *40*). By providing running wheel and time-limited food in the home cage (Fig. 3A), mice rapidly developed core symptoms of AN, including body weight decline (Fig. 3B), voluntary food restriction (Fig. 3C), increased voluntary wheel running (Fig. 3D), and shortened dropout days [defined as the number of days required to reach a 25% reduction in body weight; (*12*)] (Fig. 3E). We tagged VTA-projecting LPBN neurons with a retrograde fluorescent tracer and recorded their activity in LPBN containing brain slices from ABA mice or their food-restricted (FR) controls (Fig. 3F). The excitability of the VTA-projecting LPBN neurons, as measured by the number of action potentials elicited by current injections, was significantly increased in ABA mice compared with that in FR mice (Fig. 3, G to I). To test whether the silence of this circuit can ameliorate the ABA symptoms, we targeted it by tetanus toxin light-chain protein (TeNT) (Fig. 3J). Unexpectedly, silencing the LPBN-VTA circuit improved the retention rate (Fig. 3K), accompanied by improved body weight loss and increased food intake in ABA mice during periods of food restriction (Fig. 3, L to N). No changes in running wheel activity were observed between TeNT and mCherry mice during baseline and restriction periods (fig. S2, B and C), suggesting that exercise activity is unlikely to be the cause of the improved retention rate observed in our experiment. Overall, the improvement in the retention rate was most likely due to increased food intake during restriction periods. Because TeNT silenced the circuit throughout all phases (baseline and food restriction period) of ABA, we even observed increased baseline body weight (fig. S2A). Therefore, we normalized body weight by their baseline body weight (Fig. 3L). To specifically inhibit the LPBN-VTA circuit during the food restriction period, we targeted it using G_i_ virus (fig. S2D). Consistent with the TeNT results, inhibiting the LPBN-VTA circuit through daily administration of CNO during the food restriction period improved the retention rate of ABA mice (fig. S2E). The body weight (fig. S2G) and food intake (fig. S2H) in the G_i_ mice during food restriction periods exhibited the same increasing trends as those observed in the TeNT mice, with no change in baseline body weight (fig. S2F). Thus, we found that LPBN-VTA circuit exhibited greater excitability in ABA mice than in the FR mice. Silencing or inhibition of this circuit improved the retention rate of the ABA mice.

**Fig. 4.**
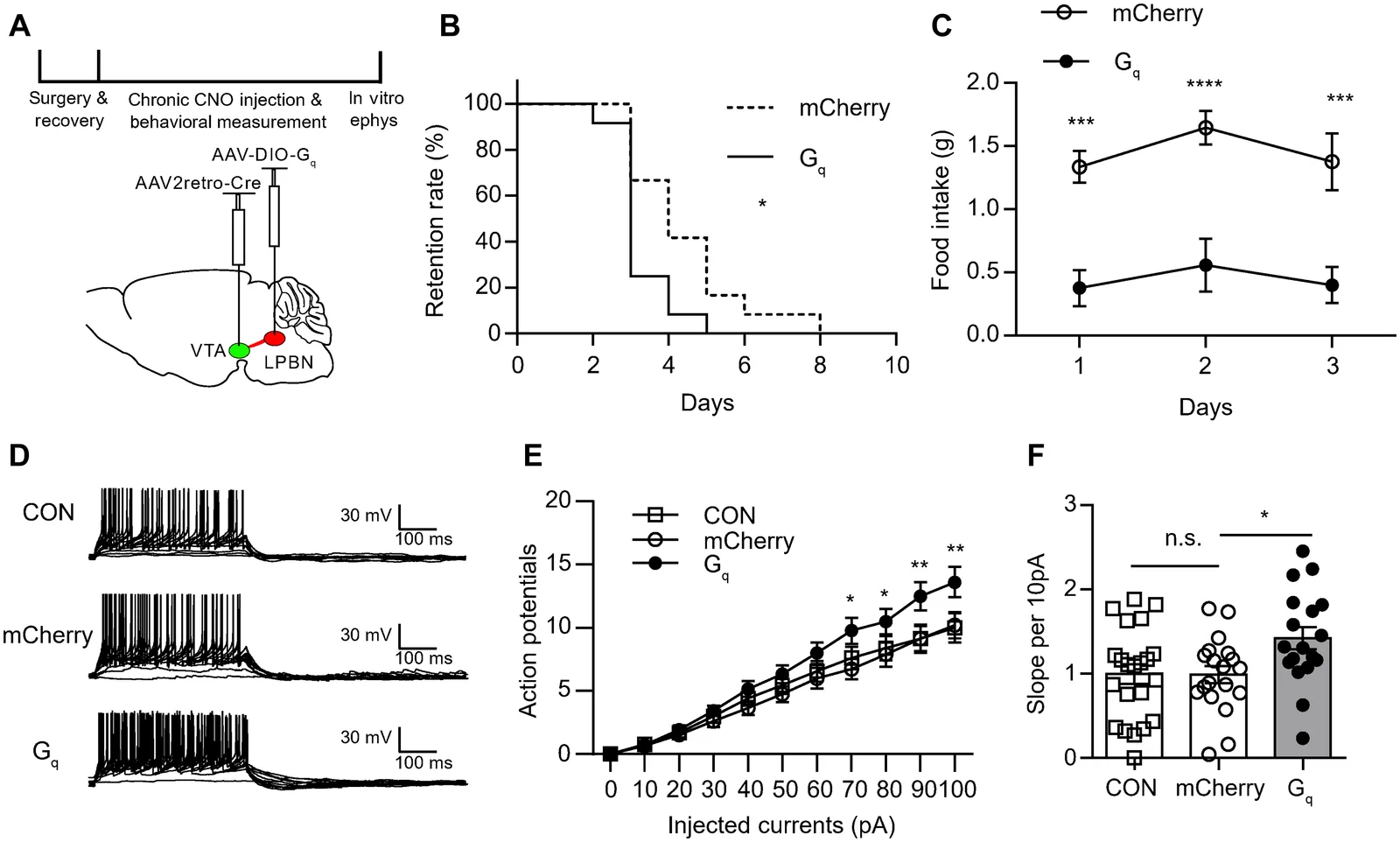
Chronic activation of LPBN to VTA behaviorally and physiologically mimics ABA mice. (**A**) Timeline of chronic activation of LPBN to VTA circuit. For the behavioral alteration tests (B and C), mCherry and G_q_ mice were subjected to FR protocol and received daily CNO injections during food restriction period. For the physiological tests (D to F), in addition to mCherry and G_q_ mice, an additional group (CON) of mice received mCherry injections but were fed ad libitum. (**B**) Chronic activation of LPBN to VTA exacerbated retention rate in G_q_ mice. (**C**) Daily CNO injections decreased food intake in G_q_ mice. (**D**) Example trace of excitability of VTA-projecting LPBN neurons among three groups. (**E**) Chronic activation of LPBN to VTA circuit increased excitability of VTA-projecting LPBN neurons. (**F**) Slope of excitability. No significant different between CON and mCherry. G_q_ significantly increased slope than mCherry.

To further support the interpretation that the LPBN-VTA circuit is causally involved in driving anorexia-like pathology, we examined whether chronic activation of the LPBN-VTA circuit in FR mice mimics the physiological and behavioral alterations observed in ABA mice. After the expression of G_q_ or mCherry in VTA-projecting LPBN neurons, mice underwent the same food restriction protocol as FR and received daily CNO injections. Body weight and food intake were monitored daily until the day of dropout when whole-cell recording was performed to assess the physiological consequences of the chronic activation (Fig. 4A). Chronic activation of LPBN-VTA circuit exacerbated the decline in the retention curve (Fig. 4B). Furthermore, chronic activation of this pathway substantially decreased food intake (Fig. 4C). Both outcomes mirrored those key characteristics shown in the ABA, indicating a sufficient role of the LPBN-VTA circuit in the development of ABA. Similar to ABA mice, chronic activation of the circuit increased the excitability of VTA-projecting LPBN neurons in FR mice (Fig. 4, D to F). There was no significant difference in excitability between ad libitum fed and FR mice (Fig. 4F), suggesting that the increased excitability observed in ABA mice is specific to the pathological state. Chronic activation of this circuit resulted in no alterations in running wheel activity in naïve mice (fig. S2I), further ruling out the involvement of this circuit in excessive wheel-running during the ABA development. Given that either the LPBN-VTA inhibition (Fig. 1K) or silencing (Fig. 3N) increased food intake and that neither chronic activation (fig. S2I) nor silencing (fig. S2C) of this circuit altered wheel running activity, the improved retention rate of ABA mice may be attributed to increased food intake during the food restriction periods.

### Glutamatergic inputs from LPBN to VTA indirectly inhibit dopamine release in the medial NAc shell

**Fig. 5.**
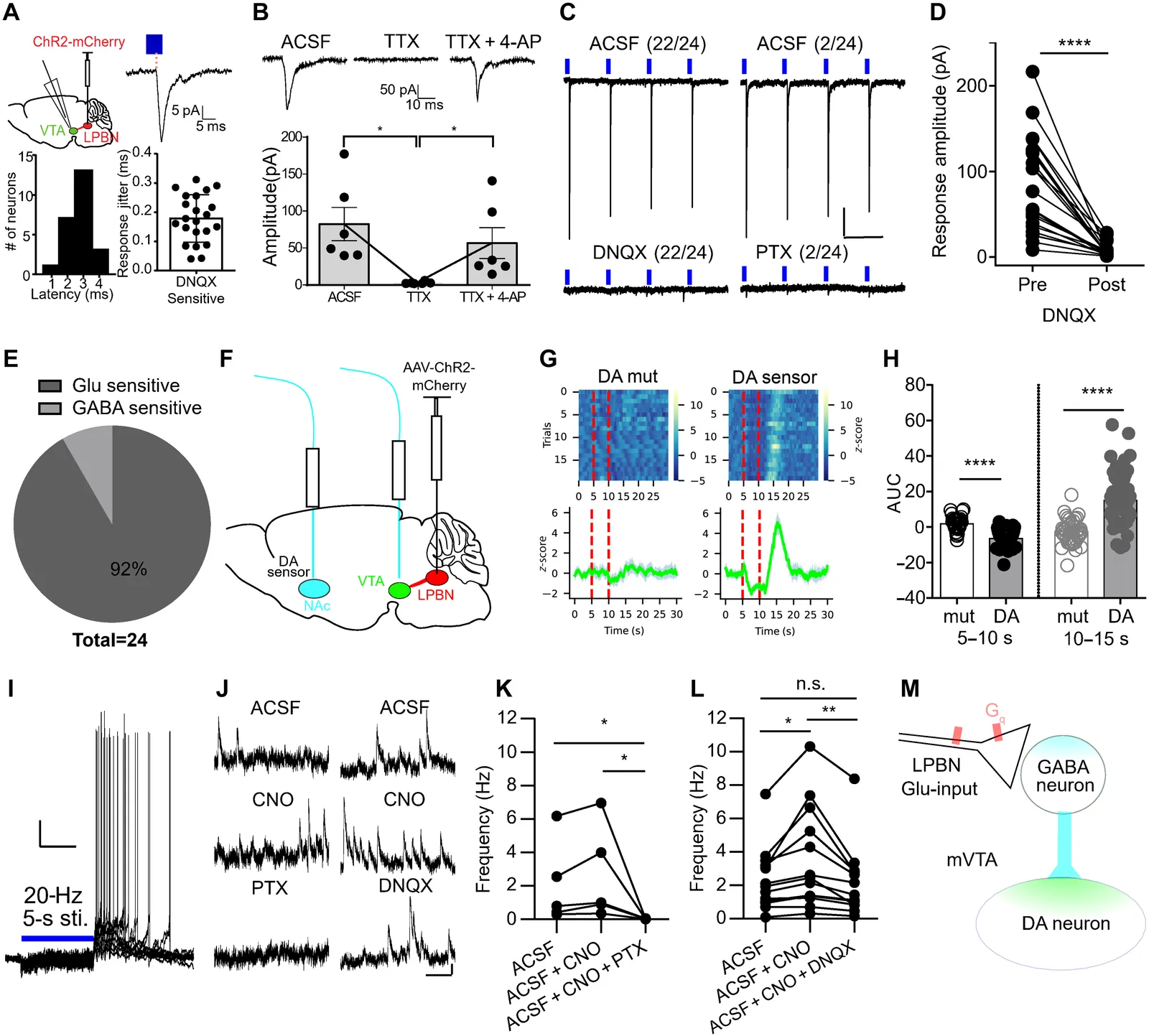
Glutamatergic inputs from LPBN to VTA indirectly inhibit DA neurons through GABA interneurons. (**A**) Top left: Schematic illustration of virus injection and electrophysiology recording strategy. Top right: Example trace of short-latency neurons. Bottom left: Histogram of latency distribution. Bottom right: Jitters of the latency. (**B**) The short latency response evoked by circuit activation revealed monosynaptic connection. (**C**) Example traces showing the light evoked currents in VTA neurons (top). Scale: 25pA, 1 s. (**D**) Light evoked current amplitudes in the VTA neurons before and after the application of the AMPAR antagonist DNQX. (**E**) Pie chart of glutamatergic and GABAergic connections. (**F**) Schematic of monitoring DA sensor fluorescence changes in the NAc during the activation of the LPBN-VTA circuit. (**G**) Example heatmaps and *z*-scored fluorescence showing DA release alterations in the medial NAc aligned to the activation of the LPBN-VTA circuit (red dashed line) in DA sensor–expressing mice (right) versus their control (DA mut mice, left). (**H**) DA release pattern in the NAc during the activation of the LPBN-VTA circuit. AUC, area under curve. (**I**) Example traces showing that 20-Hz 5-s stimulation hyperpolarized the membrane potential (blue line) and induced rebound firing upon termination of the LPBN-VTA circuit activation. Scale: 10 mV, 2.5 s. (**J**) Representative sIPSC traces recorded from the VTA DA neurons. Scale: 10 pA, 100 ms. (**K**) GABA_A_ receptor antagonist PTX completely blocked the sIPSCs recorded from VTA DA neurons. (**L**) Activation of the LPBN-VTA circuit by CNO increased sIPSC frequency recorded from VTA DA neurons. The increased sIPSC frequency returned to baseline following application of the glutamate antagonist DNQX. (**M**) Schematic diagram showing the functional connection between LPBN and VTA DA neurons.

To investigate the functional connection of the LPBN-VTA, we recorded neurons in the VTA slices containing ChR2-expressing afferent fibers from the LPBN (Fig. 5A). To identify the monosynaptic projection from the LPBN to the VTA, we only recorded neurons within short-latency (less than 5 ms) responding currents (Fig. 5A) (*41*). Consistent with a monosynaptic connection, tetrodotoxin (TTX) blocked these light-induced short-latency currents, which recovered in the presence of the potassium channel blocker 4-AP (Fig. 5B). These results suggest that the projection from the LPBN to the neurons with short-latency currents is monosynaptic. We applied receptor antagonists to further determine whether these monosynaptic currents were glutamatergic or GABAergic. We found that the currents of most neurons (22 of the 24 neurons) were blocked by the glutamate receptor antagonist DNQX (Fig. 5, C and D), with the currents of two neurons blocked by the γ-aminobutyric acid type A (GABA_A_) receptor antagonist (Fig. 5C). In DNQX-sensitive neurons, short latencies (2.75 ± 0.14 ms, Fig. 5A) and small onset jitter (0.18 ± 0.017 ms, Fig. 5A) were consistent with monosynaptic connections (*42*). Similar to previous findings, our results showed that 92% of the monosynaptic projections from the LPBN to the VTA were glutamatergic (Fig. 5E) (*43*).

Previous studies suggested that activation of projections from the VTA to the medial NAc shell could rescue the retention rate in ABA rats (*23*), implicating a role of the medial NAc shell in the development of the ABA model. We measured dopamine release in this region following the LPBN-VTA activation by combining optogenetic stimulation of LPBN terminals in the VTA with fiber photometry to detect dopamine responses in the medial NAc shell (Fig. 5F). Activation of mainly glutamatergic LPBN-VTA projections initially decreased the dopamine release in the medial NAc shell, suggesting that the activation suppressed the activity of medial VTA DA neurons indirectly via GABAergic neurons (*44*). These results were consistent with previous reports using an activity-dependent targeting strategy [targeted recombination in active populations (TRAP)] to activate the LPBN-VTA projection (*44*). Unexpectedly, a rebound dopamine release was observed within seconds after cessation of the stimulation (Fig. 5, G and H). In contrast, dopamine release increased in the lateral NAc shell during activation of the LPBN-VTA circuit (fig. S3, A and B) and increased firing of lateral VTA DA neurons in vitro (fig. S3F), suggesting that activation of the LPBN-VTA differentially modulated the medial and lateral VTA DA neurons that innervate the NAc shell, as previously reported (*43*). The same dopamine release pattern was observed in female Balb/c mice (fig. S3, C to E, I, and J, and table S1), suggesting that such neural connectivity is conserved across sexes, ages and mouse strains.

In accordance with the in vivo dopamine release pattern in the medial NAc, we found that activation of LPBN ChR2-expressing fibers onto medial VTA DA neurons produced hyperpolarization and cessation of the stimulation induced rebound firing in acute brain slices (Fig. 5I). To further confirm the indirect GABA interneuron inhibition on DA neurons, we expressed a G_q_ virus in VTA-projecting LPBN neurons using the dual viral strategy described above. We recorded the spontaneous inhibitory postsynaptic currents (sIPSCs) from the medial VTA DA neurons (fig. S3H). sIPSCs were completely blocked by the GABA_A_ receptor antagonist picrotoxin (Fig. 5, J and K), confirming that the sIPSCs onto DA neurons were GABA dependent. Application of CNO onto the slice increased the frequency of sIPSCs in the medial VTA DA neurons, with no change in the amplitude (fig. S3G). The increased sIPSC frequency returned to baseline after the application of the AMPA/Kainate receptor antagonist DNQX (Fig. 5, J and L), suggesting that the effect of LPBN stimulation on DA neurons was indirect. These results confirmed our hypothesis that activation of the VTA-projecting glutamatergic LPBN neurons inhibits the VTA DA neurons via activation of GABA interneurons (Fig. 5M).

### Decreased VTA DA neuron activity and BK channel current in ABA mice

**Fig. 6.**
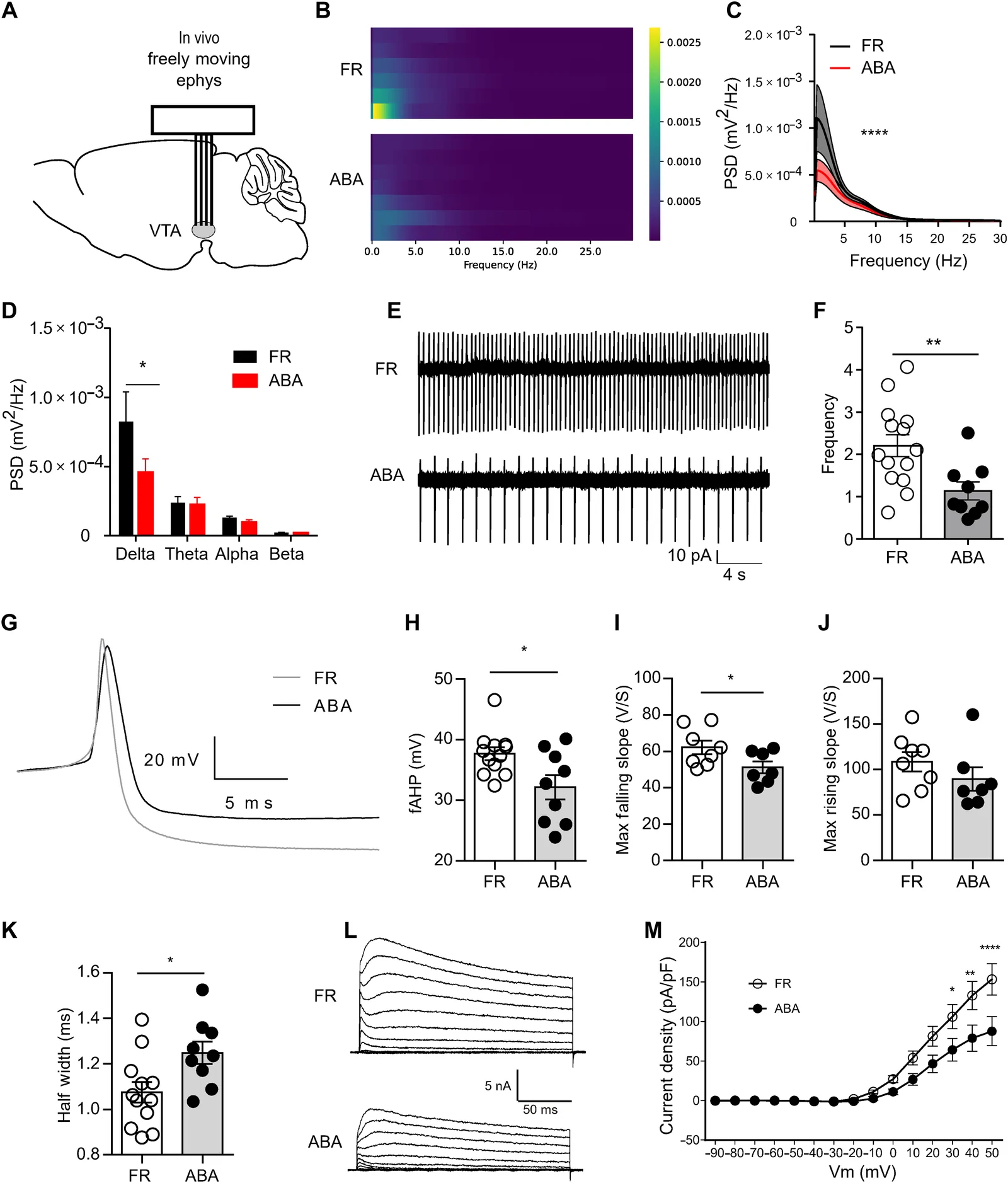
Decreased VTA DA neuron activity and BK channel currents in ABA mice. (**A**) Schematic of in vivo electrophysiology. A tetrode electrode was implanted in the VTA. Signals were recorded in the freely moving animals after recovery from surgery. (**B**) Heatmap of power spectrum density (PSD) of in vivo recordings from FR and ABA mice. (**C**) Power spectrum density analysis of in vivo recordings from ABA and FR mice. (**D**) Decreased delta-band power spectrum density in ABA mice. (**E**) Example traces of spontaneous firing in VTA DA neurons in vitro. (**F**) Spontaneous firing rates of VTA DA neurons. (**G**) Example action potential traces from VTA DA neurons of ABA and FR mice. (**H**) Fast-decaying afterhyperpolarization (fAHP). (**I**) Maximum falling slope. (**J**) Maximum rising slope. (**K**) Half width of action potentials. (**L**) Example traces of paxilline-sensitive BK currents in ABA and FR mice. (**M**) BK channel current density from VTA DA neurons of ABA and FR mice.

Because the VTA projecting neurons in the LPBN were hyperactive in ABA mice (Fig. 3) and DA neurons were indirectly inhibited by GABA interneurons upon LPBN-VTA stimulation (Fig. 5), we hypothesized that the activity of VTA DA neurons from ABA mice might be decreased. To verify this hypothesis, we implanted electrodes into the medial VTA to perform in vivo multichannel recordings. After recovery, we recorded the local field potential signals from the VTA in freely moving mice. Spectral analysis indicated that the low-frequency delta-band power was decreased in ABA mice compared with that in FR mice (Fig. 6, A to D). Previous studies indicated that the prominent rhythmic 2- to 5-Hz firing patterns of dopamine neurons in the VTA (*45*) could be the source of low-frequency oscillations (delta band) in the VTA (*46*). Thus, the decreased low-frequency oscillations observed in the ABA mice presumably represent a reduction in VTA DA neuron activity. Supporting this, slice electrophysiology data showed that the firing rate of VTA DA neurons was reduced in the ABA mice using cell-attached recordings (Fig. 6, E and F). Chronic activation of the LPBN-VTA circuit mimicked decreased firing rate in VTA DA neurons observed in ABA mice (fig. S4, F and G), supporting the hypothesis that electrophysiological changes in VTA neurons under ABA arise from the LPBN-VTA hyperactivation. Although there was no significant difference in current-evoked excitability or hyperpolarization-activated currents (*I*_h_) between dopamine neurons of ABA and FR mice (fig. S4, A to E), we observed differences in action potential waveforms obtained from whole-cell recordings (Fig. 6G). Fast-decaying afterhyperpolarization (fAHP) and maximum slope of the falling phase were notably decreased in the ABA mice (Fig. 6, H and I). No change was observed in the maximum slope of the rising phase (Fig. 6J). The half-width of the action potential was increased in the ABA mice (Fig. 6K). The above alterations indicated a reduction in large-conductance calcium- and voltage-dependent potassium channels (BK channels) (*47*). We observed the decreased BK current density of VTA DA neurons in the ABA mice from the macroscopic BK current recordings (Fig. 6, L and M).

### Activation of the BK channel rescues both DA neuron firing and retention rate in ABA mice

**Fig. 7.**
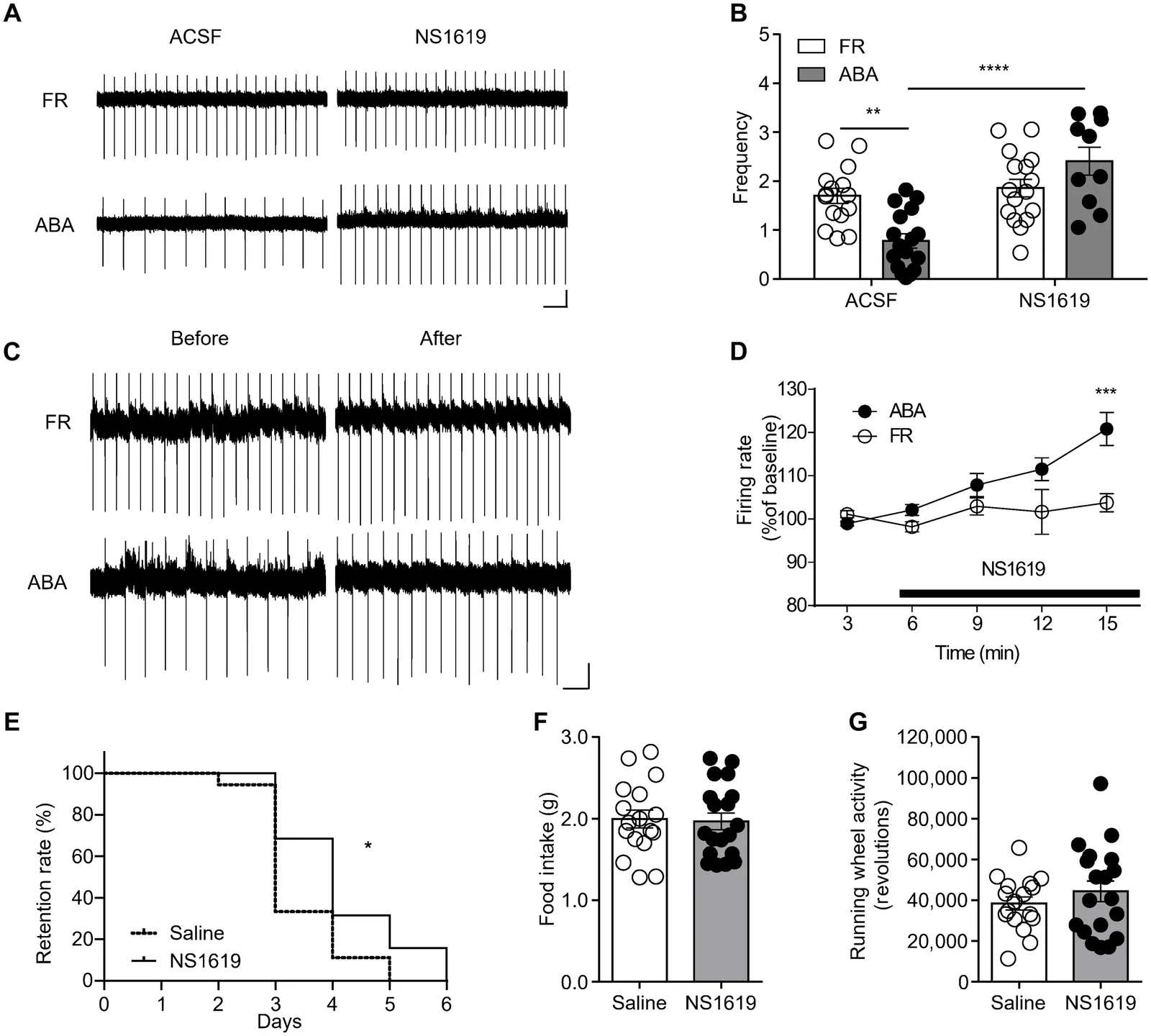
BK channel activation rescues DA neuron firing and retention rate in the ABA mice. (**A**) Example traces showing the effect of the BK channel activation on the DA neuron firing rate in ABA mice in a between-subjects design. Scale: 10 pA, 1 s. (**B**) The effect of BK channel agonist on the firing rate in a between-subjects design. (**C**) Example traces showing the effect of BK channel activation on the firing rate in a within-subjects design. Scale: 10 pA, 1 s. (**D**) The effect of BK channel agonist on the firing rate changes (% of baseline) in a within-subjects design. (**E**) The effect of intra-VTA BK channel agonist infusion on the retention rate. (**F**) The effect of intra-VTA BK channel agonist infusion on food intake. One mouse in saline group was removed due to reaching the endpoint criterion. (**G**) The effect of intra-VTA BK channel agonist infusion on voluntary running wheel activity.

To explore the potential therapeutic target, we activated BK channels in VTA DA neurons to test whether this could rescue the decreased firing rate as previously reported (*47*). DA neurons in ABA mice exhibited a significantly decreased firing frequency compared with those in FR mice, while the BK channel agonist NS1619 rescued the reduced firing frequency in the ABA mice in cell-attached recordings in vitro in both the between-group design (Fig. 7, A and B) and the within-group design (Fig. 7, C and D). This suggests that the hypoactivation of VTA DA neurons in ABA mice was induced by the dysfunction of the BK channel. To verify the role of the BK channel in ABA mice, we delivered the BK agonist NS1619 into the VTA daily during the food restriction period. The rescued retention rate was observed in the ABA mice (Fig. 7E and fig. S5). However, there was no significant difference in food intake or running wheel activity during the food restriction period (Fig. 7, F and G), suggesting that other unknown mechanisms contribute to the BK agonist-mediated improvement.

## DISCUSSION

### Activation of the LPBN-VTA suppresses feeding and promotes self-reinforcement

Because AN is characterized by abnormal eating patterns, it is crucial to investigate the role of a potentially related neural circuit in feeding behaviors. Using DREADDs, we have shown that the activation of VTA-projecting LPBN neurons led to suppression of food consumption in naïve mice, whereas inhibition of the circuit increased cumulative food intake in fasted mice, indicating the involvement of the LPBN-VTA circuit in transmitting satiety signals and suppressing food consumption (*34*). The PBN receives afferents from both the hypothalamus (*48*) and nonhypothalamus (*49*) regions and thus transmits the satiety signal. Specific activation of either source of the afferents to the LPBN can suppress food consumption by ∼50% (*19*, *49*, *50*). In our study, however, mice exhibited a remarkable 85% reduction in food consumption during their dark cycles. The notable effect of feeding suppression observed by activating VTA-projecting LPBN neurons suggests that these neurons possibly integrate satiety information from the hypothalamus (*19*, *50*) and other nonhypothalamic nuclei (*16*, *49*, *51*). The robust anorexic effect of the LPBN-VTA circuit may contribute to the high mortality rate observed in ABA mice as these mice continued to run and failed to consume sufficient food during the food available period, resulting in life-threatening weight loss within a week (*52*).

Given the robust anorexic effect, we therefore further investigated whether activation of the LPBN-VTA circuit could reinforce food restriction behaviors using RTPP and optical ICSS. Intriguingly, we demonstrated that transient activation of the circuit was reinforcing, whereas prolonged activation or inhibition of the circuit caused aversion. Although activation of the LPBN-VTA increased chamber entries in the RTPP assay, animals did not increase the total time spent in the stimulated compartment. Instead, stimulation elicited that frequent but brief visits suggest that circuit activation promotes approach behavior without supporting sustained occupancy. Animals robustly worked to obtain stimulation in an operant task, indicating that the manipulation has reinforcing properties at the level of action selection and motivation. However, the absence of conditioned place preference suggests that circuit activation does not confer stable positive contextual value. Together, these findings support the interpretation that this DA-related circuit preferentially encodes incentive motivation or action invigoration rather than hedonic reward. Consistent with contemporary models of dopamine function, the stimulation appears sufficient to reinforce goal-directed actions while failing to generate persistent affective or contextual reinforcement (*37*). Such dissociation between operant reinforcement and place-based preference has been observed in DA manipulations that enhance “wanting” or salience without engaging “liking” mechanisms or long-term value learning. We therefore propose that activation of this circuit engages a nonhedonic motivational process, potentially reflecting transient incentive salience, rather than classical reward encoding. Intermittent overengagement of this circuit in the ABA model could bias behavior toward persistent restriction and/or hyperactivity by reinforcing nonhedonic motivational drives and by producing lasting circuit adaptations, a mechanism that plausibly contributes to the etiology and persistence of AN.

There are two possible explanations for the self-reinforcing effect of transient circuit activation. First, this effect has previously been observed in the activation of VTA glutamate or glutamate/dopamine co-expressing neurons (*35*, *36*). The neuronal fibers from the LPBN may indirectly innervate these neurons via GABA interneurons in the VTA. The activity of these neurons rebounded immediately after the cessation of the LPBN-VTA activation, which may contribute to the transient appetitive behaviors. Second, VTA-projecting LPBN neurons may have heterogeneous populations of neurons encoding positive and negative valence. For instance, LPBN neurons receiving the paraventricular nucleus of the hypothalamus (PVH) inputs encode positive valence, whereas CGRP-expressing neurons encode negative valence, both of which contribute to the suppression of feeding behaviors (*19*, *53*). The net activation effect of these neuronal populations can result in a distinct pattern of neural activity that is responsible for the transient, self-reinforcing effect. Testing these two possibilities is an open area for future research. In any case, activation of the LPBN-VTA circuit showed both suppressive effects on food intake and self-reinforcing effects on operant behaviors, suggesting its potential role in the context of ABA.

In addition to our observation, activation of the PVH to the LPBN suppresses feeding and encodes positive valence in calorie-depleted mice (*19*), which also meets two criteria we proposed. A similar phenomenon was observed in a study in which stimulation of the VTA-DA neuron to the dorsal raphe nucleus (DRN) 5-HT neuron circuit resulted in appetitive behaviors while suppressing food intake in ABA mice (*22*). These observations support that the two criteria we proposed could be used to identify ABA-related circuits because patients with AN perceive food restriction behaviors as more reinforcing (*14*). Thus, the pathology of AN could involve an abnormal activation or maladaptation of these circuits, triggered by genetic susceptibility and environmental factors, complying with the DA-centered hypothesis. A particularly intriguing finding of this study is the distinct behavioral divergence elicited by different temporal patterns of circuit manipulation. While transient activation of the LPBN-VTA circuit is robustly reinforcing, its chronic activation is highly aversive and behaviorally recapitulates the core features of the ABA model. We propose that this dual valence reflects a critical neurobiological mechanism underlying the stage-dependent clinical progression of AN. Clinical evidence indicates that the early stages of AN are often characterized by goal-directed, highly reinforcing restrictive behaviors, wherein patients derive a pathological sense of control or anxiety relief from fasting. The positive reinforcement observed during acute, transient stimulation of the LPBN-VTA circuit may closely model this initial behavioral acquisition phase. However, as restricted feeding and weight loss become established, the circuit undergoes an allostatic shift into the persistent hyperactive state observed in our ABA mice. Our data demonstrate that this chronic hyperactivation drives a severe, aversive state that suppresses food intake and promotes hyperactivity, a transition likely mediated by the sustained, indirect inhibition of downstream dopamine neurons via VTA GABAergic interneurons. Thus, this circuit may act as a critical mechanistic switch. An initial transient activation reinforces the early stages of fasting behavior, which then leads to a chronic, self-sustaining hyperactive state that drives the compulsive, late-stage pathogenesis of AN. To map the precise longitudinal trajectory of this switch, tracking the firing pattern transitions of the circuit during specific behavioral micro-episodes (such as precise feeding approaches) across the multiday ABA paradigm would be an exciting future direction.

### Bidirectional modulation of the LPBN-VTA circuit prevents and mimics anorexia phenotypes

Given that the LPBN-VTA circuit is involved in food suppression and reinforcement behaviors, we further investigated the role of this circuit in the ABA mice. VTA-projecting LPBN neurons in ABA mice exhibited increased excitability compared with those in control mice, suggesting that the circuit is hyperactivated in ABA mice. Consistently, chronic activation of this pathway induced key phenotypes observed in ABA, supporting that the LPBN-VTA circuit is causally involved in the development of anorexia-like pathology. Furthermore, suppressing or silencing the LPBN-VTA circuit rescued the retention rate in ABA mice, indicating the necessity of LPBN-VTA signaling in the ABA.

Activation of the VTA projections to the medial NAc shell can rescue the anorexic phenotype in ABA rats (*23*), indicating that DA plays a pivotal role in the ABA. Based on this evidence, we thus identified the functional connection between the LPBN and VTA DA neurons by monitoring neuronal activity, and dopamine fluctuations in the medial NAc shell immediately after stimulating the LPBN-VTA circuit. In addition, we found medial-lateral differences in NAc shell dopamine dynamics, indicating the existence of subregional heterogeneity in the NAc, which is an important direction for future investigation. Our results suggest that glutamatergic LPBN neurons indirectly innervate VTA DA neurons via GABA interneurons, consistent with a previous observation from TRAP experiments (*44*). Therefore, the improved retention rate following suppression of the LPBN-VTA circuit in ABA mice is likely due to the disinhibition of VTA DA neurons mediated by GABA interneurons. Decreased spontaneous firing rate of VTA DA neurons was observed in our study when the LPNB-VTA pathway was chronically activated. This is in line with observations that dopamine D2/D3 receptor binding in the anteroventral striatum increased in patients with AN (*24*), while DA metabolites decreased in their cerebrospinal fluid (*25*). Both findings suggest hypoactivity of the DA system (*15*). Furthermore, activation of VTA neurons can rescue the retention rate of ABA (*23*, *54*).

Our findings dovetail with recent work showing that distinct VTA dopamine circuits can powerfully shape specific feeding phases (*30*).They identified a peri-LC–to–VTA pathway that acted as a critical regulator of hedonic eating by controlling the duration of food consumption. Activation of peri-LC to VTA circuit during consumption phase suppressed palatable food intake mediated by local VTA GABA neurons, which is mechanistically similar to our observations. The transient incentive salience induced by the LPBN-VTA circuit activation may be the distinct mechanism underlying ABA pathology. Despite the anatomical and behavioral similarities, we confirmed that the circuit under our investigation was confined to the LPBN-VTA and is not attributable to off-target viral expression in adjacent areas (fig. S1B).

To investigate the neurobiological mechanisms underlying activity-based anorexia (ABA), we chose adolescent female BALB/c mice due to their documented susceptibility to hyperactivity and weight-loss phenotypes in ABA paradigms and its relatively anxiety-prone behavioral profile, traits that mirror key vulnerability factors in human AN (*12*, *55*). Balb/c mice exhibit robust weight loss and shortened dropout days under ABA conditions compared with some other strains, making them useful for detecting behavioral and physiological effects of food-restriction–induced hyperactivity and for pharmacological manipulations of underlying circuits. Besides genetic background, ABA susceptibility is reported to be affected by sex and age (*12*, *55*, *56*), although their direction and magnitude depend on specific protocol parameters (*55*). To address these sources of variability and assess generalizability, we conducted key experiments in both adolescent female Balb/c and C57BL/6 mice (table S1). The core circuit mechanisms identified in the present study were replicated across both genetic backgrounds, indicating that the observed neural circuit regulation of ABA-related behaviors is not strain specific and likely reflects a conserved mechanism underlying susceptibility and resistance to maladaptive starvation-driven activity.

A two-stage model of AN characterized by opposite changes in dopamine function was proposed (*57*). In the first stage, caloric restriction combined with exercise triggers an increase in dopamine, facilitating the establishment of weight-loss behaviors. Conversely, in the second stage, chronic self-starvation leads to a reduction or impairment of dopamine, leading to behavioral inflexibility and entrenchment of AN behaviors. It potentially explains the complexity and some seemingly contradictory findings regarding the DA system in ABA or AN (*22*, *23*, *26*, *56*). For instance, a recent study found a higher spontaneous firing rate in VTA DA neurons of ABA mice (*58*), which could reinforce the weight-loss behaviors as matched to the first stage of the two-stage model.

### BK channel as a potential target for the treatment of AN

The BK channel is a critical molecular determinant in the control of neuronal excitability, playing an important role in regulating action potential duration, firing frequency, and neurotransmitter release. Our results demonstrated that restoring BK channel function in VTA DA neurons improved the retention rate of ABA mice, suggesting that BK channels were involved in the ABA model. Notably, the dysfunction of BK has been reported to reduce tonic firing while enhancing the phasic firing of VTA DA neurons (*47*, *59*). These findings are consistent with clinical observations that AN exhibits low DA metabolite levels in CSF and heightened DA-related responses to cues (*10*, *24*, *25*, *60*). However, the involvement of BK in ABA in the present study should be approached with greater caution when extending it to AN for several reasons. First, the alleviation of ABA is not attributable to improved food intake or running wheel activity in our results, suggesting that other unknown mechanisms are involved. Second, the BK gene, located on chromosome 10 in humans, has not been reported to be associated with AN in genome-wide analysis (*61*, *62*). Nonetheless, considering the beneficial effects of BK agonists in ABA mice, it is worthwhile to invest further research into elucidating the relationship between BK and ABA. In summary, the findings of the present study have elucidated the critical role of the LPBN-VTA circuit and the potential channel pathology in the disorder, which may serve as a key to the development of drug treatments and intervention strategies for AN.

## MATERIALS AND METHODS

### Animals and ABA modeling

Female Balb/c mice aged 3 weeks were ordered for ABA modeling. To rule out differences in strain or sex, both female Balb/c and male C57BL/6 mice were used for behavior tests and neural circuit dissection. Mice subjected to the ABA procedure were individually housed in cages with controlled light and temperature on a 12-hour inverted light/dark cycle, light on at 7:00 p.m. All procedures were performed in accordance with the Animal Care Committee of East China Normal University (m20190235). For detailed ABA modeling, see Supplementary Methods.

### Virus injection and optical fiber implantation

The diluted virus was infused into the target site using Nanojector III. Optical fibers were implanted into the target sites after virus infusion. See Supplementary Methods for details on virus injection and fiber implantation.

### Optogenetics and fiber photometry

The ChR2 or DA sensor was expressed on the VTA-projecting LPBN neurons or NAc neurons, respectively, by targeted viral infusion. Optical stimulation fibers were implanted in the VTA, whereas detection fibers were implanted in the NAc. Timing was aligned using a pulse generator module (Thinkerbiotech, China). See Supplementary Methods for more details.

### Measurement of food intake

Mice were individually housed after the virus injection surgery. CNO or saline was injected intraperitoneally into satiated or fasted mice. Body weight and cumulative food intake were monitored daily. See Supplementary Methods for more details.

### Real-time place preference

Mice were observed using a camera positioned above a customized box with two chambers connected by a tunnel. The location of mouse was tracked in real-time using MATLAB-based programs. When a mouse entered the activation chamber, a computer triggered a stimulation pattern to a laser source. For excitation experiments, viruses were injected into the bilateral LPBN, and a fiber was implanted into the VTA. After recovery, mice explored the apparatus for 10 min to establish initial preferences with connecting fibers. On the following days, mice received various light (473 nm) stimulation patterns when entering the stimulation chamber. The time spent in each chamber was recorded for analysis. For inhibition experiments, different viruses were injected, and yellow light (589 nm) was used to inhibit neural circuits. The time spent and the number of entries in each chamber were recorded for further analysis. See Supplementary Methods for more details.

### Dropout test in the AN animal

Retrograde Cre-expressing viruses were injected into the VTA while Cre-dependent viruses were injected into the LPBN to inhibit or silence the LPBN-VTA circuit, using either G_i_ or TeNT. The circuit was inhibited by CNO daily during the development period of ABA in G_i_-expressing mice. For the BK agonist dropout test, a cannula was implanted into the VTA as previously described (*63*). Mice received daily injections of the BK agonist or vehicle before food during the development period of ABA. Body weight below 75% of their baseline was the criterion for dropout (*12*). See Supplementary Methods for more details.

### Optical self-stimulation test

Mice were injected with either the ChR2 or control virus in the LPBN, and a fiber was implanted in the VTA. After recovery, they were habituated to an operant chamber (Lafayette Instrument) for 3 days. Mice were trained to nose poke the screen for a milkshake reward to ensure unbiased participation. During activation, nose poking triggered brief stimulations of the LPBN-VTA circuit, but no milkshake was delivered. Mice underwent six training sessions followed by a no-stimulation phase where nose poking no longer activated the circuits. The number of active nose pokes was counted daily for further analysis. See Supplementary Methods for more details.

### In vivo electrophysiology

Custom-made electrodes were made as previously described (*64*) and then were implanted into the VTA of ABA or FR mice. After the ABA procedure, electric signals were recorded from the freely moving mice on the third day of food restriction. MATLAB was used to filter the signals and extract the local field potentials. See Supplementary Methods for more details.

### In vitro electrophysiology

In vitro electrophysiology studies were conducted as previously described (*63*). Briefly, brains of anesthetized mice were perfused, and brain slices containing the VTA or LPBN were prepared. Slices were incubated in artificial cerebrospinal fluid (ACSF) solutions and later transferred to a recording chamber. Fluorescent cells were visualized using a microscope, and electronic signals were collected using the MultiClamp 700B amplifier and 1440A digitizer. For cell-attached recordings, spontaneous firing was recorded for 10 min. The effects of the BK agonist NS1619 were tested by introducing it into the recording chamber. Whole-cell recordings were used to study *I*_h_ currents, excitability, and action potentials. To investigate the LPBN-VTA circuit, ChR2 virus was injected into the LPBN, and neurons in the VTA were recorded under blue light (473 nm) stimulation. To isolate voltage-dependent potassium currents, 4-AP and TTX were added to the ACSF solution, and sulpiride was used to block D2R signaling. Outward currents were evoked by depolarizing voltage steps, and the BK antagonist paxilline was used to identify BK currents. A Python-based program was used to analyze action potential waveforms. See Supplementary Methods for more details.

### Data analysis

All values are expressed as means ± SEM. n.s., no significant; *P < 0.05; **P < 0.01; ***P < 0.001; and ****P < 0.0001. Data were checked with normal distribution test and the homogeneity of variance between groups before any parametric test. The *t* test was used when there were two groups in the experiment. Analysis of variance (ANOVA) was used for multiple group comparisons. The paired-sample *t* test or repeated measures ANOVA was used for the within group experiments. The independent-sample *t* test or one-way ANOVA was used for the between group experiments. Bonferroni or Šidák test was used to correct multiple comparisons in ANOVA. Wilcoxon matched-pairs signed-rank test or Gehan-Breslow-Wilcoxon test was used for data that did not meet the criterion of parametric test. Log-rank (Mantel-Cox) test was used for the retention rate analysis. Excel (Microsoft), Python, MATLAB, and Prism (GraphPad) were used for data analysis. A summary of statistics is provided in table S2.

## Supplementary Materials

### The PDF file includes:

Supplementary Text Figs. S1 to S5 Tables S1 and S2 Legends for movies S1 and S2 References
