# Supplementary material for: A pontomesencephalic-mesolimbic circuit modulates activity-based anorexia
Source: Sci Adv. 2026 Sep 23;12(39):eaee1476. doi: 10.1126/sciadv.aee1476 (PMC13600181; doi:10.1126/sciadv.aee1476)
Supplement: Supplementary file 1 [file sciadv.aee1476_sm.pdf]

Science Advances
AAAS

Supplementary Materials for

A pontomesencephalic-mesolimbic circuit modulates activity-based anorexia

Beibei Peng *et al.*

Corresponding author: Shuai Liu, sliu@psy.ecnu.edu.cn

Sci. Adv. **12**, eaee1476 (2026)
DOI: 10.1126/sciadv.aee1476

The PDF file includes:

Supplementary Text
Figs. S1 to S5
Tables S1 and S2
Legends for movies S1 and S2
References

Other Supplementary Material for this manuscript includes the following:

Movies S1 and S2

Supplementary Text

Supplementary Methods:

ABA model. In the ABA model, female Balb/c mice arrived at the facility at postnatal day 21 (P21). After acclimatizing to their new environment for one week, each mouse was introduced to a cage equipped with a running wheel and provided with food and water ad libitum for an additional week. Two groups were used in our studies: ABA mice were given progressive food access and unlimited access to a running wheel at the onset of dark-cycle as previously reported (*65*). Specifically, mice were subjected to 4 hours of food restriction on the first day, which was then reduced to 3 hours on subsequent days. FR mice had the same food restriction schedule as the ABA group, but their running wheel was locked. We focused on food intake behavior rather than the effects of voluntary exercise based on our proposed criteria. Therefore, we used FR as our consumption control instead of an exercise control group (mice with unlimited access to food and running wheel) to probe its effects (*12*, *23*, *56*), even though exercise was reported to play roles in ABA models(*58*, *66*). Body weight, food intake, and running wheel counts were recorded daily. Mice were excluded from the experiments (dropout) when they lost 25% of their initial body weight, which provided a measure of survival(*12*).

Virus injection and optic fiber implantation. C57BL/6 male or Balb/c female mice were anesthetized with pentobarbital sodium (i.p., 80 mg/kg) and given ketoprofen for pain relief (subcutaneous, 5 mg/kg). Mice were mounted in the stereotaxic apparatus (RWD), and a craniotomy was conducted at the region of interest. Virus was delivered to the target site at 1 nl/s rate using a pressure injection system (Nanojector III) with a volume of 200 nl per site. All viruses were diluted to 2-3 $\times 10^{12}$ copies per ml.

The coordinates of injection sites were: lateral parabrachial nucleus (-5.20 mm anteroposterior (AP), ±1.40 mm mediolateral (ML) and -3.35 mm dorsoventral (DV)), ventral tegmental area (- 3.20 mm AP, ±0.50 mm ML, -4.60 mm DV), nucleus accumbens lateral shell (LatNAc, +0.98 mm AP, -1.80 mm ML (left), -4.92 mm DV), nucleus accumbens medial shell (MedNAc, +1.50 mm, + 0.55 mm ML (right), -4.70mm DV). The VTA and MedNAc required a 10˚-degree angle to implant the fiber with a 200 μm working distance; otherwise, the fiber was implanted perpendicularly with a 200 μm working distance.

Optogenetics and fiber photometry. We injected the ChR2 viruses into LPBN, DA sensor (rAAV-CAG-GRAB-DA1h-WPRE-hGH pA) or its control (rAAV-CAG-GRAB-DA mut-WPRE-hGH pA) into MedNAc and LatNAc. Stimulation fiber (0.37 NA, 200 μm diameter) was implanted bilaterally in the VTA at an angle of 10˚. Detection fiber (0.50 NA, 200 μm diameter) was implanted in MedNAc and LatNAc. The MedNAc required 10˚ to implant the detection fiber. After full expression of the virus, we tested the DA signal in the NAc with the stimulation of the LPBN-VTA circuit. We combined the fiber photometry system and the optogenetic system with a signal modulator that could align the timing of these two systems. Mice were anesthetized with pentobarbital sodium throughout the recording period. Each mouse received 30 sweep trials. Each sweep had an interval of 30 seconds, which contained 3 phases: baseline phase, stimulation phase, and post-stimulation phase. In the baseline phase, we monitored the DA signal in NAc for 5 seconds without stimulation of the LPBN-VTA circuit. In the stimulation phase, the LPBN-VTA circuit was activated by 473 nm wavelength light through the fiber implanted in the VTA at 20 Hz for 5 seconds, and the DA signal was collected through the detection fiber implanted in the NAc. In the post-stimulation phase, the stimulation was terminated, and the DA signal in the NAc was collected for 20 seconds.

Behavioral tests

Measurement of food intake. All mice were singly housed following virus injection. Mice were handled daily and received three mock intraperitoneal (i.p.) injections one week prior to the initiation of food intake measurement. Studies were performed in the mouse home cage with standard chow ad libitum during the dark cycle. For the Gq experiments, satiated mice received saline (i.p.) at the beginning of the dark cycle on day 1. CNO (1mg/kg, i.p.) was delivered at the same time on day 2. Body weight was monitored daily, and 4 hours of cumulative food intake was monitored after i.p. injection. After one week of the injections, post-fasting refeeding studies were conducted. Animals were fasted for 16h prior to measurement. 4-hour cumulative food intake was monitored in the same way. For Gi experiments, satiated mice received CNO (i.p.) at a dose of 5 mg/kg. The dose of CNO used in the tests (1mg/kg for Gq and 5mg/kg for Gi) does not affect the food intake behaviors according to previous studies(*50*). 3-hour cumulative food intake was monitored after i.p. injection of CNO in satiated mice. Post-fasting refeeding studies were conducted one week later. Mice were fasted for 16h prior to measurement. 4 hours cumulative food intake was monitored after i.p. injection of CNO.

Real-time place preference. Mice were captured by a camera mounted on the top of the customized box (two separate chambers with different contexts connected with a tunnel). The centroid of the mouse was calculated in real-time by our customized programs edited by Matlab, thus we could monitor the location of the mouse in real-time (see the movie in the supplementary material). Once the mouse entered the activation chamber, the computer sent a signal to our signal modulator through the USB

cable and then triggered the pre-edited stimulation pattern to the laser source via a BNC cable.

In excitation experiments, the virus (AAV2/9-hSyn-hChR2(H134R)-mCherry for excitation animals or AAV2/9-hSyn-mCherry-WPRE-pA for control animals) was injected into the bilateral LPBN, and the fiber (0.37 numerical aperture (NA), 200 μm diameter) was implanted into the VTA at an angle of 10° bilaterally at least 3 weeks after the virus injection surgery. After 1 week of recovery, mice acclimated to the apparatus for 10 minutes by connecting the fibers without activation on day 1. The apparatus was cleaned with 75% ethanol between each mouse to eliminate the residual odors left by the previous mouse. On day 2, mice received 1 Hz stimulation with blue light (473 nm wavelength) when they entered the stimulation chamber; the test period lasted 10 minutes per mouse. In the following 8 days, mice received different stimulation patterns (2 Hz, 4 Hz, 8 Hz, 10 Hz, 20 Hz, and then 1 Hz, followed by two different burst-like stimulations, 4B and 10 B, meaning stimulation with 4 or 10 Hz for 1 second and then no light for 3 seconds) when they entered the stimulation chamber. The stimulation side was counterbalanced between mice. The time spent on the stimulation side and the number of entries were recorded for further statistical analysis.

In inhibition experiments, the virus (rAAV-hSyn-eNpHr3.0-EYFP-hGH-pA for inhibition mice or rAAV-hSyn-EYFP-WPRE-pA for control mice) was injected into the LPBN, and the fiber was implanted in the same way as in the excitation experiments. Mice were acclimated to the apparatus on day 1. On the second day, mice received 2s on / 2s off yellow light (589 nm wavelength) in the VTA to inhibit the LPBN-VTA neural circuit when it entered the designated chamber. Mice in the stimulation side were counterbalanced. The time spent in the stimulation side and the number of entries were recorded for further statistical analysis.

Dropout test in the ABA. To inhibit the LPBN-VTA circuit in a specific time window, we injected the AAV2/8-hSyn-DIO-hM4D(Gi)-mCherry or AAV2/8-hSyn-DIO-mCherry into the LPBN and AAV2/2Retro-CMV_bGI-Cre-EGFP-pA into the VTA. Mice (Gi and mCherry mice) were subjected to the ABA procedure mentioned above after full expression of the virus. CNO (5 mg/kg, i.p.) was delivered daily, 30 minutes prior to the onset of the dark cycle during the food restriction period of ABA procedures. Body weight and food intake were monitored daily until all mice reached the dropout criterion (a 25% loss of their body weight) (*12*). Alterations in body weight, food intake, and running wheel activity during periods of food restriction were measured two days after the initiation of the restriction, at which point only a small fraction of the mice had dropped out, ensuring that there were enough mice for analysis.

To achieve permanent silencing of the LPBN-VTA circuit, we injected the virus (AAV2/9-HEF1a-DIO-mCherry-P2A-TetTox-WPRE-pA or AAV2/9-hSyn-DIO-mCherry-WPRE-pA into LPBN and AAV2/2Retro-CMV_bGI-Cre-EGFP-pA into VTA), which expressed the tetanus neurotoxin in the targeted neurons to cleave essential proteins involved in the exocytotic machinery, resulting in inhibition of neurotransmitter release(*67*). The same ABA procedure as in the Gi experiments was used to investigate the necessity of the circuit in the development of the ABA model.

To investigate the effect of the BK agonist (NS1619) in the VTA, we implanted the guide cannulas (26 gauge; RWD 62064) bilaterally in the VTA. The ABA model was established using the same procedure as described above. Mice received a daily microinjection of the BK agonist 30 minutes prior to the onset of the dark cycle. Vehicle (saline containing 10% DMSO) or BK agonist (1 mM dissolved in 10% DMSO) was infused bilaterally into the VTA at a volume of 0.5 μl/side at a rate of 0.1 μl/min. The microinjectors were left in place for an additional 3 min after the injection. The mice were then

returned to their home cage.

Optical self-stimulation test. Mice were injected with ChR2 or control virus into the LPBN and the fiber was implanted into the VTA. After full expression of the virus, mice were habituated to the touch screen version of the operant chamber (Lafayette Instrument) for 3 days. A customized program was edited in the Animal Behavior Environment Test (ABET) software (Lafayette Instrument). We trained mice to achieve a palatable milkshake (Yazoo Strawberry UHT milkshake; Friesland Campina) by touching two screens in the operant chamber for three days. After this procedure, a brief stimulation (2 pulses at 20 Hz, 473 nm wavelength) replaced the natural reward (milkshake) when mice touched one of the two screens (the active touchscreen). Mice received 6 consecutive training sessions (one session per day). After the activation phase, mice received a session in which nose-poking the active screen did not trigger the stimulation of the circuit.

Electrophysiology

In vivo electrophysiology. 16-channel tetrode electrodes were made according to the procedures described in the previous study (*64*). Briefly, four single electrodes were twisted into one tetrode by using a reversible stepper motor accurately controlled by an ESP-32 microcomputer (33 μm diameter, Nicochrome, California Fine Wire). Electrodes of the tetrode were bound tightly together using a 420°C hot wind. Then the tetrodes were connected to a custom-made circuit board, and then connected to the pre-amplifier in the in vivo electrophysiology recording system (Plexon).

We implanted the electrode into the VTA of ABA or FR mice. After recovery from the surgery, we established the ABA model. We recorded the electronic signals in the freely moving ABA and FR mice

on the third day of the food restriction period at the same circadian time as in vitro tests (zeitgeber time (ZT) 15; light on at ZT 0). Raw signals were overviewed by visual inspection, and only noise-free channels were used for analysis. Matlab was used to filter the signal and extract the local field potential. Fast Fourier transform was applied to the time series of the filtered signals, and the power spectrum was examined. We measured the alteration of delta (0.5-4 Hz), theta (5-8 Hz), alpha (8-12 Hz), and beta (12-30 Hz) bands(*68*) after the ABA procedure.

In vitro electrophysiology. After the mice were fully anesthetized with pentobarbital sodium, and then perfused with the ice-cold perfusion solution containing (in mM): 250 Glycerol, 2.5 KCl, 2 $MgCl_2$, 1.25 NaH_2PO_4, 10 HEPES, 25 $NaHCO_3$, 2 $CaCl_2$, 11 Glucose, saturated with 95%O_2/5% CO_2. Mice were decapitated, and brains were rapidly extracted. Brain slices containing VTA (horizontal, 250 µm thickness) or LPBN (coronal, 250 µm thickness) were prepared in the perfusion solutions using the vibratome (Leica Microsystems). The sectioned slices were transferred into an incubation chamber containing the ACSF solutions (in mM): 126 NaCl, 1.6 KCl, 1.1 NaH2PO4, 1.4 MgCl2, 2.4 CaCl2, 26 NaHCO3, and 11 Glucose, saturated with 95%O2/5% CO2 at 32°C for at least 1 hour before transfer to the recording chamber. For cells expressing the fluorescent signal, we visualized the fluorescent positive cells with fluorescent-matched excitation/emission filters mounted in the microscopes. Otherwise, cells were visualized with the infrared differential interference contrast video microscope.

We used the MultiClamp 700B amplifier (Molecular Devices) and the 1440A digitizer (Molecular Devices) to collect the electronic signal. Borosilicate glass with filaments was pulled with P-2000 pullers (Sutter Instrument) to generate the appropriate electrode (2-7 MΩ).

For cell-attached recording, we used the internal solutions containing (in mM): 140 K D-Gluconate,

5 KCl, 10 HEPES, 0.2 EGTA, 2 NaCl, 2.5 Mg-ATP, 0,25 Na-GTP, 0.2% (w/v) biocytin. After forming the giga-Ohm seal, hold the cell with the leakage currents around 0 pA. Spontaneous firing was recorded for 10 minutes before the cell was ruptured. To investigate the effect of the BK agonist (NS1619) on the ABA mouse, we designed within and between group experiments by washing NS1619 (10 μM) into the recording chamber. Whole-cell recording lasted at least 5 minutes to allow the biocytin to equilibrate into the cytoplasm. After equilibration, the electrode was gently removed.

We used K D-Gluconate solutions to test the Ih currents, excitability, and action potentials in the ABA or FR mice. To test Ih current, we used a series of hyperpolarizing step voltage pulses (from -60 mV to -120 mV) with an increment of -10 mV by holding the cell at -70 mV for 3s. To test the excitability of the neurons, we injected 400 ms depolarizing step currents (from 0 pA to 350 pA with an increment of 50 pA for VTA neurons and 0 pA to 100 pA with an increment of 10 pA for LPBN neurons). Whole-cell recordings lasted at least 5 minutes to allow the biocytin to equilibrate into the cytoplasm. Dopamine neurons were identified by the Ih current and biocytin labeling as previously described (*63*).

To investigate the projection type of the circuit from the LPBN to the VTA in vitro, we injected the ChR2 virus into the LPBN and recorded the neurons in the VTA. We used the 1440A digitizer (Molecular Devices) to control the stimulating blue light with precise timing. CsCl-based internal solution containing (in mM): 135 CsCl, 10 HEPES, 4 NaCl, 0.5 Na-GTP, 5 Mg-ATP, 1.125 MK801, 0.2% (w/v) biocytin. MK801 was used to block the NMDAR signaling (*69*). After forming the whole-cell recording, cells were held at -70 mV. Signals evoked by the stimulating blue light (4 pulses at 1 Hz per sweep, with a duration of 8s) were recorded. Each neuron was subjected to 30 sweeps. Then the AMPAR blocker DNQX (10 μM) was applied in the ACSF for 10 minutes, and light evoked signals were recorded. If light evoked signals had no response to the DNQX application, picrotoxin (100μM)

was washed into the recording chamber for 10 minutes to block $GABA_AR$ mediated response.

To isolate the voltage-dependent outward potassium currents, we added 4-AP (1 mM) and TTX (0.5 μM) to the external ACSF solutions to reduce or block the voltage-dependent potassium and sodium currents, respectively. Sulpiride (1 μM) was used to block the D2R signaling, which could contaminate the voltage-dependent outward potassium current in the VTA(*47*). After forming the whole-cell recording, cells were held at -70 mV. We compensated for the membrane and the serial resistance (60% on-line). The liquid-junction potential (approximately 10 mV in our configuration) was not corrected for all neurons. Leak currents were subtracted using a standard P/4 protocol. Outward currents were evoked by a series of 200 ms depolarizing voltage steps from -90 mV to +50 mV in 10 mV increments. After recording the outward potassium currents in the ACSF, the BK antagonist (paxilline, 10 μM) was washed into the chamber for at least 10 minutes, considering that it took time for hydrophobic molecules to penetrate into the brain slices(*70*). Paxilline-sensitive currents (BK currents) were calculated by subtracting the trace after the paxilline from the trace before the paxilline. The current density (pA/pF) was used as a readout of the functional BK channels, considering different membrane capacitances among different neurons.

To analyze the action potential waveform of each neuron, we wrote a Python-based program to extract waveform parameter data from action potential recordings. The threshold of action potential was defined as 10 V/S in the first temporal derivative of the membrane voltage(*71*, *72*). The maximum up or down slope in the rising or falling phase was defined as the maximum or the minimum of the first temporal derivative of the membrane voltage during the rising and falling phase, respectively. The fast after-hyperpolarization potential (fAHP) was defined as the difference between the minimum potentials during the falling phase and the threshold potentials. The coefficient of variation larger than 15% was

excluded from the calculation.

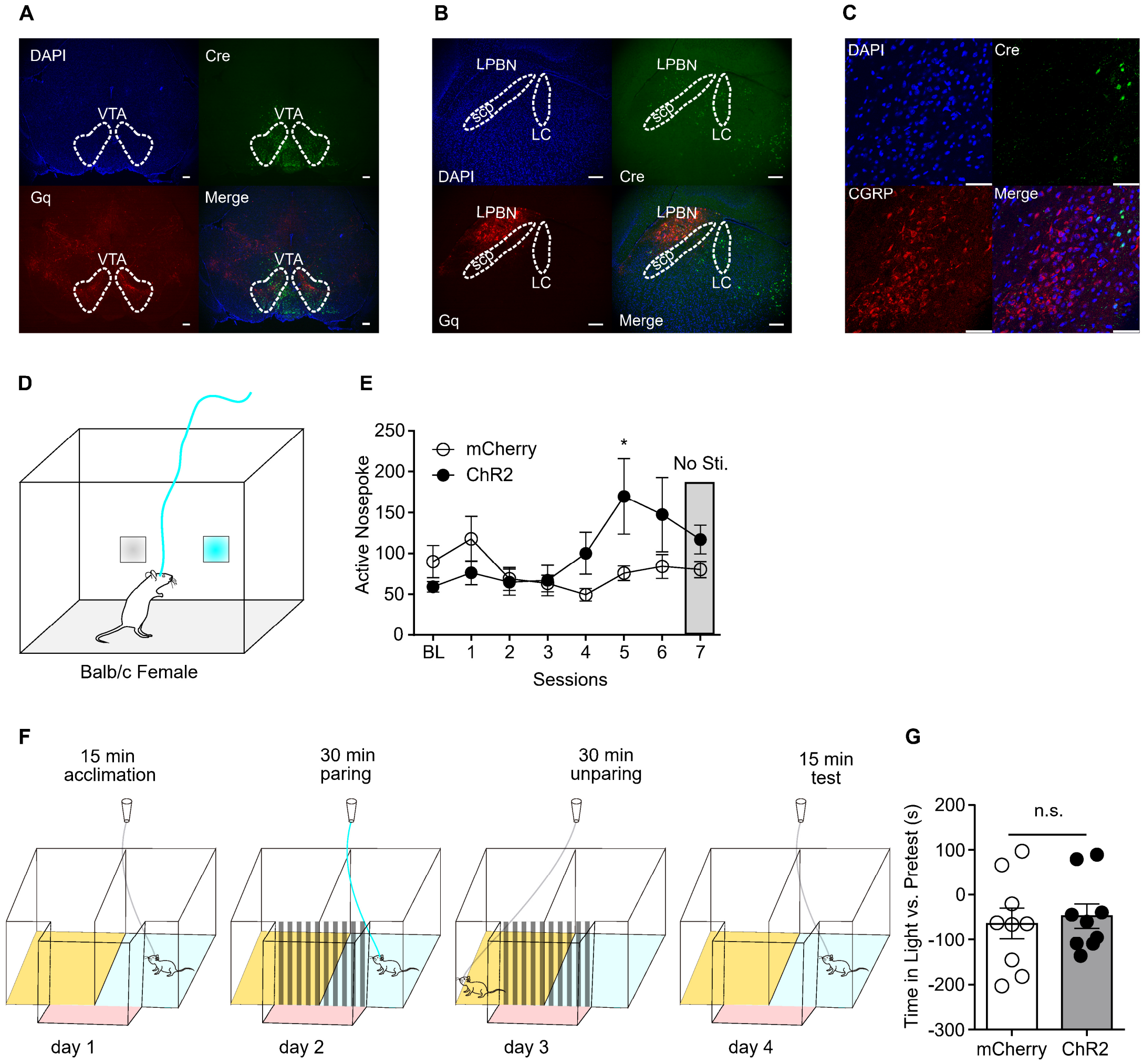

Figure S1. Demonstration of the LPBN-VTA circuit and reinforcing behaviors in Balb/c female mice.

A. Retrograde AAV Cre-expressing virus was injected into the VTA and Cre-dependent Gq virus was injected into the LPBN. Gq fibers and Cre expression were colocalized in the VTA. Scale bar: 200 µm.

B. Gq expression was confined in the LPBN. Scale bar: 200 µm.

C. VTA-projecting LPBN neurons were not colocalized with CGRP-expressing neurons (abcam, ab36001). Scale bar: 50 µm.

D. Schematic of the optogenetic self-stimulation test in Balb/c female mice.

E. ChR2 mice showed more nose-poke responses in the optogenetic self-stimulation test on the fifth day in female Balb/c mice.

F. Test procedure of optical stimulation induced conditioned place preference in C57BL/6 male mice.

G. Preference after one-session paired activation of the LPBN-VTA circuit.

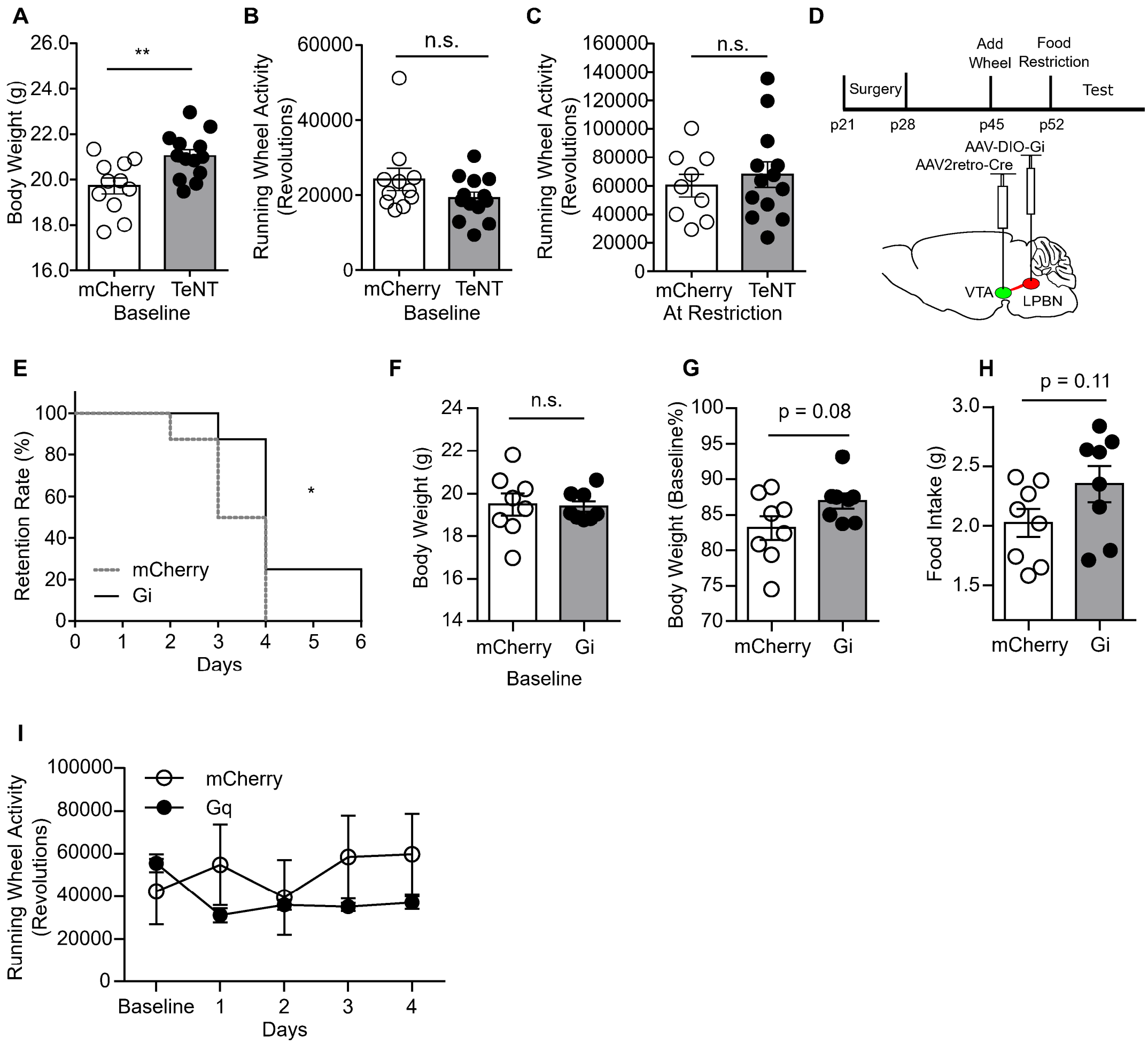

Figure S2. Silencing or inhibition of the LPBN-VTA pathway during the food restriction period improved the retention rate of ABA mice.

A. Permanent silencing of LPBN-VTA by TeNT significantly increased body weight even before ABA model.

B. Silencing LPBN-VTA had no effect on running wheel activity before initiation of ABA model.

C. No difference in running wheel activity during food restriction period in TeNT mice.

D. Schematic of injection strategy showing how to target the LPBN-VTA circuit by Gi-expressing virus.

E. Retention rate improved by chemogenetic inhibition of the LPBN-VTA circuit in ABA mice. CNO is administered daily after food restriction.

F. No difference between Gi and mCherry mice before initiation of ABA model in body weight.

G. Dropping body weight trended to alleviate after inhibition of LPBN-VTA during the food restriction period.

H. Food intake trended to increase by inhibition of the LPBN-VTA circuit in ABA mice during the food restriction period.

I. Chronic activation of LPBN to VTA circuit did not change running wheel activity.

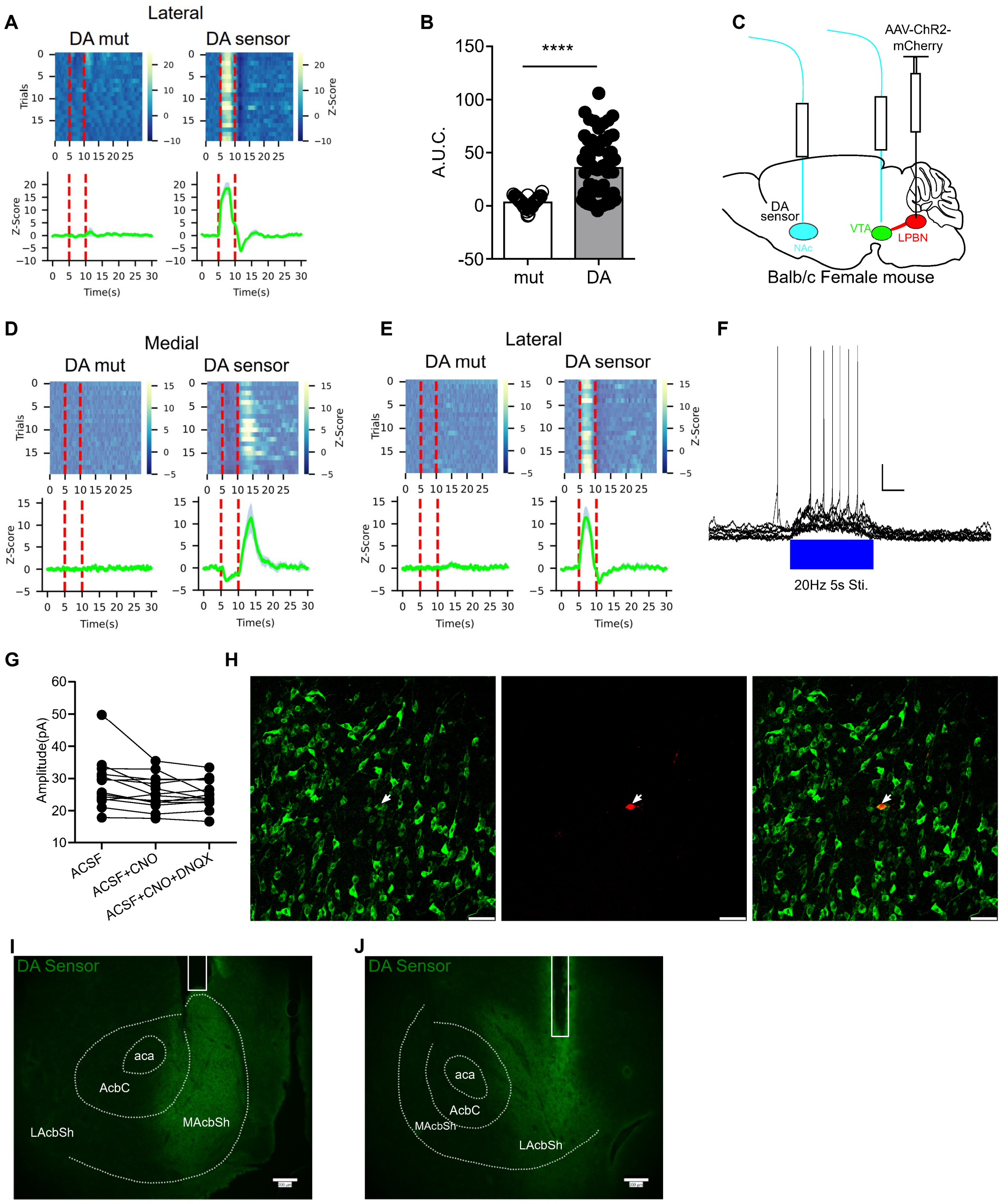

Figure S3. Characteristics of the LPBN-VTA circuit.

A. Example heatmaps and Z-scored fluorescence showing activation of the LPBN-VTA induced DA release pattern in the lateral NAc Shell in male C57BL/6 mice.

B. The area under curve (AUC) during the 5s stimulation of the LPBN-VTA, showing increased DA release in the lateral NAc shell.

C. Schematic of monitoring DA sensor fluorescence changes in the NAc during the activation of the LPBN-VTA circuit in Balb/c female mice.

D. Example heatmaps and Z-scored fluorescence showing activation of the LPBN-VTA induced DA release pattern in the medial NAc Shell in female Balb/c mice.

E. Example heatmaps and Z-scored fluorescence showing activation of the LPBN-VTA induced DA release pattern in the lateral NAc Shell in female Balb/c mice.

F. Example trace showing activation of the LPBN-VTA circuit induced firing in the lateral VTA DA neurons in male C57BL/6 mice. Scale: 10 mV,1s.

G. Chemogenetic activation of the LPBN-VTA circuit did not change the sIPSC amplitude recorded from the VTA DA neurons.

H. Post-hoc TH immunofluorescence (Sigma, T1299) was identified through loading biocytin into patch pipette. TH positive neurons (left, green) overlapped with patched cells (middle, red) indicating the patched cell was DA neuron (right). Scale: 50 μm.

I. Location of fibers and the DA sensors signal in medial NAc shell (MAcbSh). DA sensor was enhanced by EGFP antibody (ThermoFisher, MA5-15256). Scale: 200 μm.

J. Location of fibers and the DA sensors signal in the lateral NAc shell (LAcbSh). Scale: 200 μm.

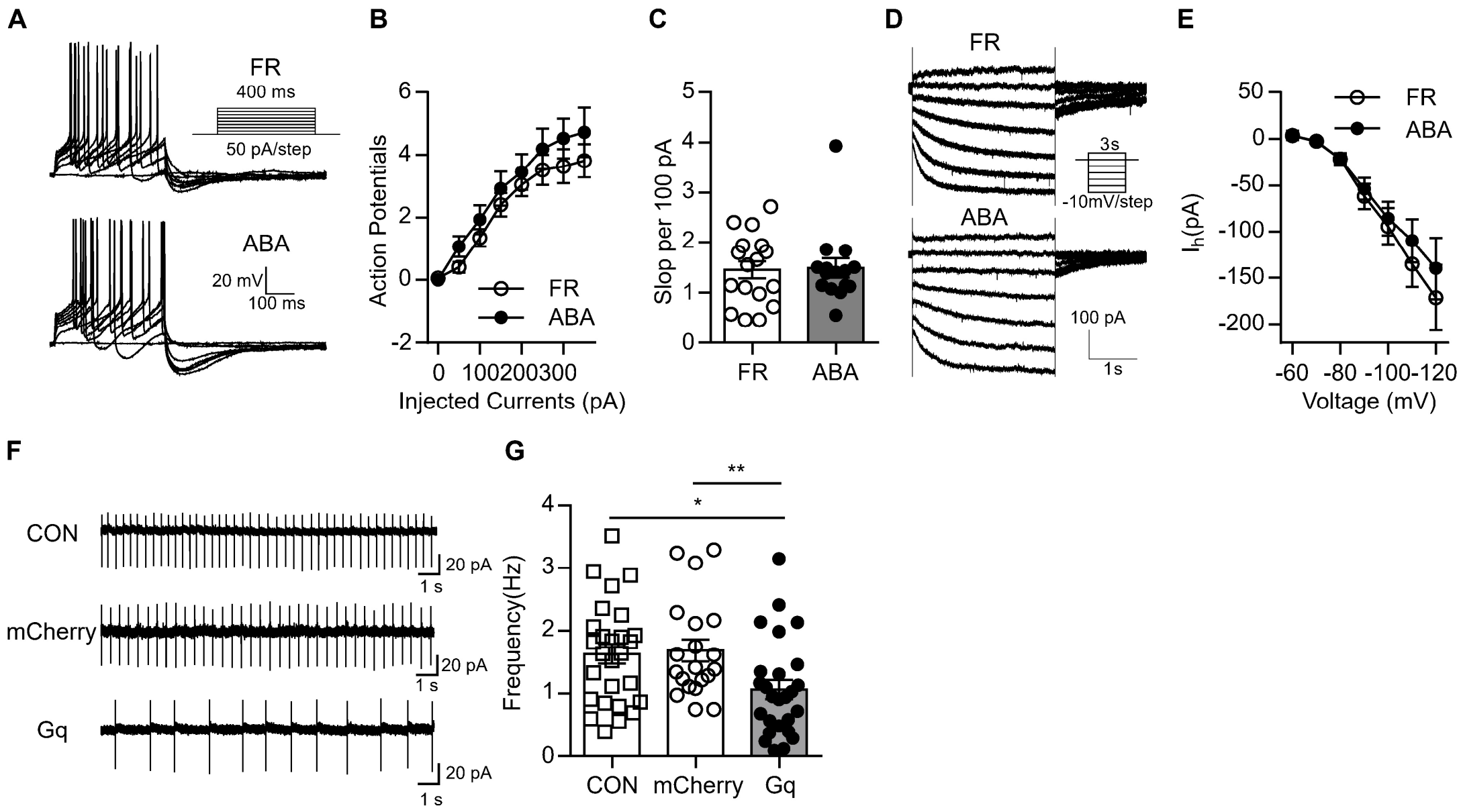

Figure S4. No alteration in the excitability or Ih currents of DA neurons in ABA mice.

A. Example traces of current injection induced action potentials of DA neurons in FR and ABA mice.

B. Number of action potentials in response to current injection.

C. The slope of the spike number per 100 pA.

D. Example Ih current traces of DA neurons in FR and ABA mice.

E. Ih currents had no change in ABA mice.

F. Example trace showing chronic activation of the LPBN to VTA pathway and its effects on the spontaneous firing of VTA DA neurons.

G. Chronic activation of the LPBN to VTA pathway decreased spontaneous firing of VTA DA neurons.

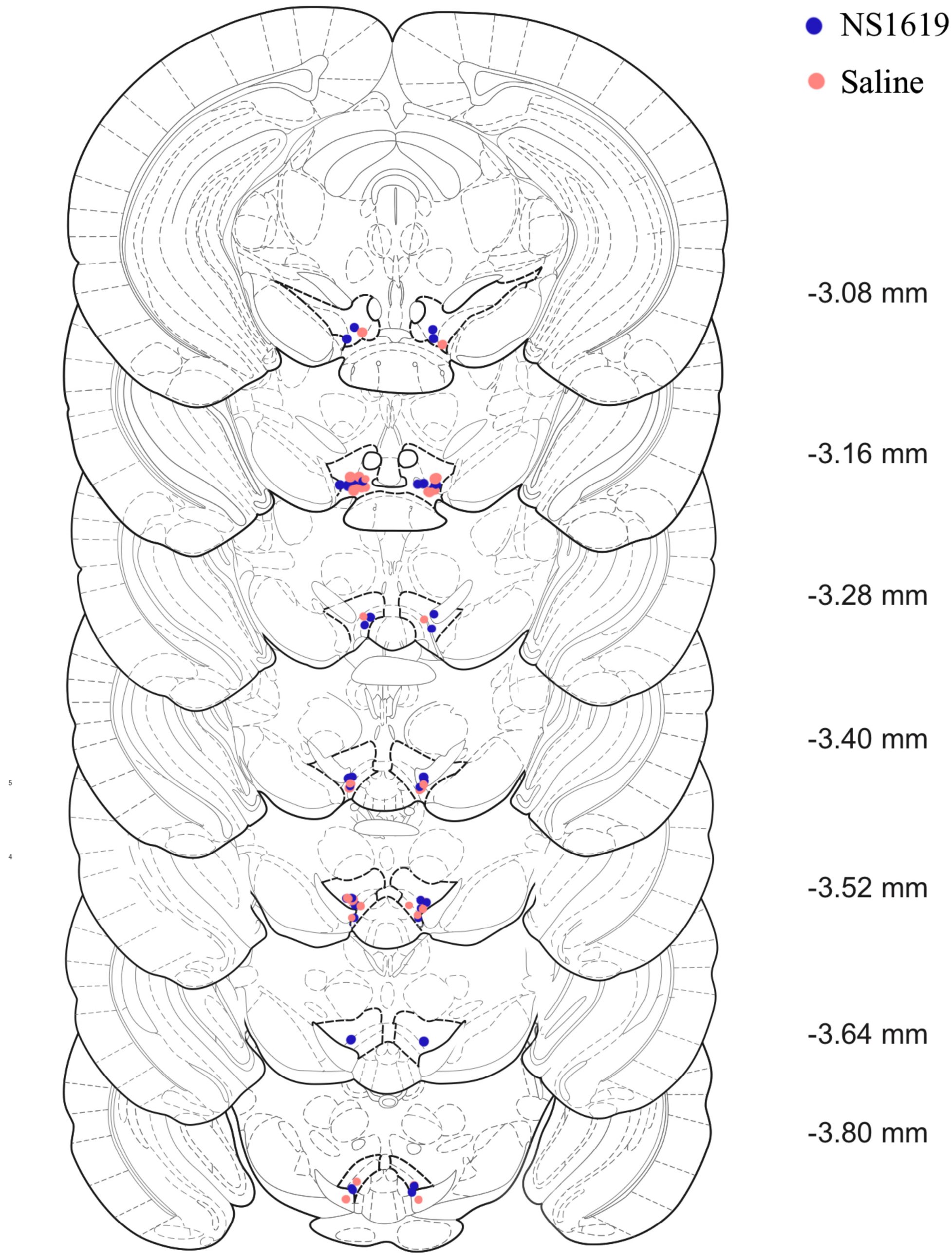

Figure S5. Injection sites of intra-VTA infusion are shown in coronal sections.

Blue dots: NS1619; red dots: Saline. Distance from bregma is shown to the right of each section (in millimeters).

Table S1. Summary table of mouse strains, sex and age

Figures	Mouse strain line	Gender	Age
Fig. 1A-K	C57BL/6	Male	8w-12w (Adolescence to Early adulthood)
Fig. 2A-J	C57BL/6	Male	8w-12w (Adolescence to Early adulthood)
Fig. 3A-N	Balb/c	Female	P38-P56 (Adolescence)
Fig. 4A-F	Balb/c	Female	P38-P56 (Adolescence)
Fig. 5A-M	C57BL/6	Male	8w-12w (Adolescence to Early adulthood)
Fig. 6A-M	Balb/c	Female	P38-P56 (Adolescence)
Fig. 7A-G	Balb/c	Female	P38-P56 (Adolescence)
Fig. S1A-E	Balb/c	Female	P38-P56 (Adolescence)
Fig. S1F-G	C57BL/6	Male	8w-12w (Adolescence to Early adulthood)
Fig. S2A-I	Balb/c	Female	P38-P56 (Adolescence)
Fig. S3A, B, F-H	C57BL/6	Male	8w-12w (Adolescence to Early adulthood)
Fig. S3C-E, I, J	Balb/c	Female	P38-P56 (Adolescence)
Fig. S4a-g	Balb/c	Female	P38-P56 (Adolescence)
Fig. S5	Balb/c	Female	P38-P56 (Adolescence)

Table S2. Summary statistics

Figure	Comparison, sample size	Statistical Test	p-value	t/W or F value, Stat
1E	mCherry vs. Gq, n = 9-10	Two-way repeated measures ANOVA	0.6938	Group effect: $F_{(1, 17)} = 0.1604$
1F	mCherry vs. Gq, n = 9-10	Two-way repeated measures ANOVA with Sidak's multiple comparisons	0.0228	Group effect: $F_{(1, 17)} = 6.264$
1G	mCherry vs. Gq, n = 10 per group	Unpaired t-test	< 0.0001	$t_{(18)} = 9.553$
1H	mCherry vs. Gq, n = 9-10	Unpaired t-test	< 0.0001	$t_{(17)} = 6.518$
1J	mCherry vs. Gi, n = 10-12	Unpaired t-test	0.6977	$t_{(20)} = 0.3941$
1K	mCherry vs. Gi, n = 10-12	Unpaired t-test	0.0428	$t_{(20)} = 2.163$
2D	mCherry vs. ChR2, n = 10 per group	Two-way repeated measures ANOVA with Sidak's multiple comparisons	0.0031	Group effect: $F_{(1, 18)} = 11.68$
2E	mCherry vs. ChR2, n = 10 per group	Two-way repeated measures ANOVA with Sidak's multiple comparisons	0.4223	Group effect: $F_{(1, 18)} = 0.6744$
2G	eYFP vs. eNpHR, n = 10 per group	Unpaired t-test	0.0380	$t_{(18)} =2.240$
2H	eYFP vs. eNpHR, n = 10 per group	Unpaired t-test	0.3866	$t_{(18)} =0.8873$
2J	mCherry vs. ChR2, n = 5-6	Two-way repeated measures ANOVA with Sidak's multiple comparisons	0.0251	Group effect: $F_{(1, 9)} = 7.197$
3B	FR vs. ABA, n = 22-29	Two-way repeated measures ANOVA with Sidak's multiple comparisons	< 0.0001	Interaction effect: $F_{(4, 196)} = 7.669$
3C	FR vs. ABA, n = 22-29	Two-way repeated measures ANOVA with Sidak's multiple comparisons	0.0462	Group effect: $F_{(1, 49)} = 4.183$
3D	Baseline vs post, n = 24	One-way repeated measures ANOVA with Sidak's multiple comparisons	0.0001	$F_{(2.287, 52.61)} = 39.44$
3E	FR vs. ABA, n = 22-29	Log-rank (Mantel-Cox) test	0.0131	$\chi^2_{(1)} = 6.157$
3H	FR vs. ABA, n = 20-21	Two-way repeated measures ANOVA with Sidak's multiple comparisons	0.0194	Group effect: $F_{(1, 39)} = 5.942$
3I	FR vs. ABA, n = 20-21	Unpaired t-test	0.0143	$t_{(39)} = 2.565$
3K	mCherry vs. TeNT, n = 11-13	Log-rank (Mantel-Cox) test	0.0048	$\chi^2_{(1)} = 7.944$
3L	mCherry vs. TeNT, n = 11-13	Unpaired t-test	0.0411	$t_{(22)} = 2.170$
3M	mCherry vs. TeNT, n = 11-13	Unpaired t-test	0.8213	$t_{(22)} = 0.2285$

3N	mCherry vs. TeNT,<br>n = 9-13	Unpaired t-test. Two mice in the mCherry group were removed due to reaching the endpoint criterion.	0.0147	$t_{(20)} = 2.669$
4B	mCherry vs. Gq,<br>n = 12 per group	Log-rank (Mantel-Cox) test	0.0198	$\chi^2_{(1)} = 5.427$
4C	mCherry vs. Gq,<br>n = 12 per group	Two-way ANOVA with Sidak's multiple comparisons	< 0.0001	Group effect: $F_{(1, 62)} = 52.88$
4E	CON vs. mCherry vs. Gq. N =18-23	Two-way repeated measures ANOVA with Tukey multiple comparisons	0.0001	Interaction effect: $F_{(20, 580)} = 2.679$
4F	mCherry vs. Gq. n =18-23	Unpaired t test	0.014	$t_{(35)} = 2.588$
5B	ACSF vs. TTX,<br>TTX vs. TTX+4-AP,<br>n = 6	Wilcoxon matched-pairs signed rank test	0.0313,<br>0.0313	W = -21,<br>W = 21
5D	Pre vs. Post,<br>n = 22	Wilcoxon matched-pairs signed rank test	<0.0001	W = -253
5H	mut vs DA (5-10s),<br>mut vs DA (10-15s),<br>n = 100	Unpaired t test	< 0.0001<br>< 0.0001	$t_{(98)} = 10.25$,<br>$t_{(98)} = 7.371$
5K	ACSF vs.<br>ACSF+CNO vs.<br>ACSF+CNO+PTX,<br>n = 5	Kruskal-Wallis test with Dunn's post hoc test	0.0018	9.637
5L	ACSF vs.<br>ACSF+CNO vs.<br>ACSF+CNO+DNQX,<br>n = 13	One-way repeated measures ANOVA with Tukey's multiple comparisons.	0.0031	$F_{(1.253, 15.04)} = 10.94$
6C	FR vs. ABA,<br>n = 6-7	Two-way ANOVA	< 0.0001	Group effect, $F_{(1, 1320)} = 53.45$
6D	FR vs. ABA in Delta,<br>n = 6-7	Two-way ANOVA with Sidak's multiple comparisons test	0.0163	Sidak's multiple comparisons test
6F	FR vs. ABA,<br>n = 9-14	Unpaired t test	0.0084	$t_{(21)} = 2.910$
6H	FR vs. ABA,<br>n = 9-12	Unpaired t test	0.0165	$t_{(19)} = 2.629$
6I	FR vs. ABA,<br>n = 7-8	Unpaired t test	0.0473	$t_{(13)} = 2.191$
6J	FR vs. ABA,<br>n = 7-8	Unpaired t test	0.2687	$t_{(13)} = 1.156$
6K	FR vs. ABA,<br>n = 9-12	Unpaired t test	0.0194	$t_{(19)} = 2.555$
6M	FR vs. ABA,<br>n = 6-11	Two-way repeated measures ANOVA with Sidak's multiple comparisons	< 0.0001	Interaction effect: $F_{(14, 210)} = 3.769$

7B	ACSF: FR vs. ABA,<br>ABA: ACSF vs. NS1619,<br>n = 10-17	Two-way ANOVA with Tukey's test	0.0002	Interaction effect: $F_{(1, 54)} = 15.58$
7D	NS1619: FR vs. ABA,<br>n = 5-11	Two-way repeated measures ANOVA with Sidak's multiple comparisons	0.0027	Interaction effect: $F_{(4, 56)} = 4.622$
7E	Saline vs. NS1619,<br>n = 18-19	Log-rank (Mantel-Cox) test	0.0174	$\chi^2_{(1)} = 5.658$
7F	Saline vs. NS1619,<br>n = 17-19	Unpaired t test	0.8601	$t_{(34)} = 0.1776$
7G	Saline vs. NS1619,<br>n = 17-19	Unpaired t test	0.3480	$t_{(34)} = 0.99516$
S1E	mCherry vs. ChR2,<br>n = 7-9	Two-way repeated measures ANOVA with Sidak's multiple comparisons	0.0025	Interaction effect: $F_{(7, 105)} = 3.416$
S1G	mCherry vs. ChR2,<br>n = 9 per group	Unpaired t test	0.7003	$t_{(16)} = 0.3919$
S2A	mCherry vs. TeNT,<br>n = 11-13	Unpaired t test	0.0069	$t_{(22)} = 22.979$
S2B	mCherry vs. TeNT,<br>n = 11-13	Unpaired t test	0.1392	$t_{(22)} = 1.534$
S2C	mCherry vs. TeNT,<br>n = 9-13	Unpaired t test	0.5513	$t_{(20)} =0.6060$
S2E	mCherry vs. Gi,<br>n = 8 per group	Log-rank (Mantel-Cox) test	0.0499	$\chi^2_{(1)} = 3.846$
S2F	mCherry vs. Gi,<br>n = 8 per group	Unpaired t test	0.8710	$t_{(14)} =0.1654$
S2G	mCherry vs. Gi,<br>n = 8 per group	Unpaired t test	0.0793	$t_{(14)} = 1.892$
S2H	mCherry vs. Gi,<br>n = 8 per group	Unpaired t test	0.1080	$t_{(14)} =1.717$
S2I	mCherry vs. Gq,<br>n = 4 per group	Two-way repeated measures ANOVA with Sidak's multiple comparisons	0.5128	Group effect: $F_{(1, 6)} = 0.4835$
S3B	mut vs. DA,<br>n = 100	Unpaired t test	< 0.0001	$t_{(98)} = 7.083$
S3G	ACSF vs. ACSF+CNO,<br>ACSF+CNO vs. ACSF+CNO+DNQX,<br>n = 14	Repeated measures one-way ANOVA with Tukey's multiple comparisons test	0.0538,<br>0.7692	Tukey's multiple comparisons test
S4B	FR vs. ABA,<br>n = 15-17	Two-way repeated measures ANOVA	0.3135	Group effect: $F_{(1, 30)} = 1.051$
S4C	FR vs. ABA,	Unpaired t test	0.8848	$t_{(30)} = 0.1461$

	n = 15-17			
S4E	FR vs. ABA,<br>n = 11-16	Repeated measures two-way ANOVA	0.5819	Group effect: $F_{(1, 25)} = 0.3111$
S4G	CON vs. Gq,<br>mCherry vs. Gq,<br>n = 20-27	Unpaired t test	0.0119,<br>0.0098	$t_{(51)} = 2.609$,<br>$t_{(44)} = 2.699$

Movie S1. Real time place preference test. The mouse decreased exposure time whereas increased entries into the blue light paired chamber (stripe), where it received the LPBN-VTA circuit activation.

Movie S2. Optical self-stimulation test. LPBN-VTA was activated upon touching the upper touchscreen (active screen) via a transient blue pulse.

REFERENCES

1. S. Zipfel, K. E. Giel, C. M. Bulik, P. Hay, U. Schmidt, Anorexia nervosa: Aetiology, assessment, and treatment. *Lancet Psychiatry* **2**, 1099–1111 (2015).

2. E. Attia, Anorexia nervosa: Current status and future directions. *Annu. Rev. Med.* **61**, 425–435 (2010).

3. F. R. E. Smink, D. van Hoeken, H. W. Hoek, Epidemiology of eating disorders: Incidence, prevalence and mortality rates. *Curr. Psychiatry Rep.* **14**, 406–414 (2012).

4. T. Ágh, G. Kovács, D. Supina, M. Pawaskar, B. K. Herman, Z. Vokó, D. V. Sheehan, A systematic review of the health-related quality of life and economic burdens of anorexia nervosa, bulimia nervosa, and binge eating disorder. *Eat. Weight Disord.* **21**, 353–364 (2016).

5. J. Treasure, S. Zipfel, N. Micali, T. Wade, E. Stice, A. Claudino, U. Schmidt, G. K. Frank, C. M. Bulik, E. Wentz, Anorexia nervosa. *Nat. Rev. Dis. Primer* **1**, 15074 (2015).

6. V. Pascoli, J. Terrier, A. Hiver, C. Lüscher, Sufficiency of mesolimbic dopamine neuron stimulation for the progression to addiction. *Neuron* **88**, 1054–1066 (2015).

7. J. Xue, D. Qian, B. Zhang, J. Yang, W. Li, Y. Bao, S. Qiu, Y. Fu, S. Wang, T.-F. Yuan, W. Lu, Midbrain dopamine neurons arbiter OCD-like behavior. *Proc. Natl. Acad. Sci. U.S.A.* **119**, e2207545119 (2022).

8. I. F. Arroteia, A. Husch, M. Baniasadi, F. Hertel, Impressive weight gain after deep brain stimulation of nucleus accumbens in treatment-resistant bulimic anorexia nervosa. *BMJ Case Rep.* **13**, e239316 (2020).

9. F. A. Cowdrey, R. J. Park, C. J. Harmer, C. McCabe, Increased neural processing of rewarding and aversive food stimuli in recovered anorexia nervosa. *Biol. Psychiatry* **70**, 736–743 (2011).

10. A.-K. Fladung, G. Grön, K. Grammer, B. Herrnberger, E. Schilly, S. Grasteit, R. C. Wolf, H. Walter, J. von Wietersheim, A neural signature of anorexia nervosa in the ventral striatal reward system. *Am. J. Psychiatry* **167**, 206–212 (2010).

11. C. E. Wierenga, A. Bischoff-Grethe, A. J. Melrose, Z. Irvine, L. Torres, U. F. Bailer, A. Simmons, J. L. Fudge, S. M. McClure, A. Ely, W. H. Kaye, Hunger does not motivate reward in women remitted from anorexia nervosa. *Biol. Psychiatry* **77**, 642–652 (2015).

12. S. J. Klenotich, M. P. Seiglie, M. S. McMurray, J. D. Roitman, D. Le Grange, P. Dugad, S. C. Dulawa, Olanzapine, but not fluoxetine, treatment increases survival in activity-based anorexia in mice. *Neuropsychopharmacology* **37**, 1620–1631 (2012).

13. C. F. Zink, D. R. Weinberger, Cracking the moody brain: The rewards of self starvation. *Nat. Med.* **16**, 1382–1383 (2010).

14. C. B. O'Hara, I. C. Campbell, U. Schmidt, A reward-centred model of anorexia nervosa: A focussed narrative review of the neurological and psychophysiological literature. *Neurosci. Biobehav. Rev.* **52**, 131–152 (2015).

15. W. H. Kaye, J. L. Fudge, M. Paulus, New insights into symptoms and neurocircuit function of anorexia nervosa. *Nat. Rev. Neurosci.* **10**, 573–584 (2009).

16. G. Williams, C. Bing, X. J. Cai, J. A. Harrold, P. J. King, X. H. Liu, The hypothalamus and the control of energy homeostasis: Different circuits, different purposes. *Physiol. Behav.* **74**, 683–701 (2001).

17. C. A. Campos, A. J. Bowen, C. W. Roman, R. D. Palmiter, Encoding of danger by parabrachial CGRP neurons. *Nature* **555**, 617–622 (2018).

18. S. Reilly, R. Trifunovic, Lateral parabrachial nucleus lesions in the rat: Neophobia and conditioned taste aversion. *Brain Res. Bull.* **55**, 359–366 (2001).

19. A. S. Garfield, C. Li, J. C. Madara, B. P. Shah, E. Webber, J. S. Steger, J. N. Campbell, O. Gavrilova, C. E. Lee, D. P. Olson, J. K. Elmquist, B. A. Tannous, M. J. Krashes, B. B. Lowell, A neural basis for melanocortin-4 receptor–regulated appetite. *Nat. Neurosci.* **18**, 863–871 (2015).

20. M. Watabe-Uchida, L. Zhu, S. K. Ogawa, A. Vamanrao, N. Uchida, Whole-brain mapping of direct inputs to midbrain dopamine neurons. *Neuron* **74**, 858–873 (2012).

21. F. Brambilla, C. S. Garcia, S. Fassino, G. A. Daga, A. Favaro, P. Santonastaso, C. Ramaciotti, E. Bondi, C. Mellado, R. Borriello, P. Monteleone, Olanzapine therapy in anorexia nervosa: Psychobiological effects. *Int. Clin. Psychopharmacol.* **22**, 197–204 (2007).

22. X. Cai, H. Liu, B. Feng, M. Yu, Y. He, H. Liu, C. Liang, Y. Yang, L. Tu, N. Zhang, L. Wang, N. Yin, J. Han, Z. Yan, C. Wang, P. Xu, Q. Wu, Q. Tong, Y. He, Y. Xu, A D2 to D1 shift in dopaminergic inputs to midbrain 5-HT neurons causes anorexia in mice. *Nat. Neurosci.* **25**, 646–658 (2022).

23. C. J. Foldi, L. K. Milton, B. J. Oldfield, The role of mesolimbic reward neurocircuitry in prevention and rescue of the activity-based anorexia (ABA) phenotype in rats. *Neuropsychopharmacology* **42**, 2292–2300 (2017).

24. G. K. Frank, U. F. Bailer, S. E. Henry, W. Drevets, C. C. Meltzer, J. C. Price, C. A. Mathis, A. Wagner, J. Hoge, S. Ziolko, N. Barbarich-Marsteller, L. Weissfeld, W. H. Kaye, Increased dopamine D2/D3 receptor binding after recovery from anorexia nervosa measured by positron emission tomography and [^{11}C]raclopride. *Biol. Psychiatry* **58**, 908–912 (2005).

25. W. H. Kaye, G. K. W. Frank, C. McConaha, Altered dopamine activity after recovery from restricting-type anorexia nervosa. *Neuropsychopharmacology* **21**, 503–506 (1999).

26. A. C. Welch, J. Zhang, J. Lyu, M. S. McMurray, J. A. Javitch, C. Kellendonk, S. C. Dulawa, Dopamine D2 receptor overexpression in the nucleus accumbens core induces robust weight loss during scheduled fasting selectively in female mice. *Mol. Psychiatry* **26**, 3765–3777 (2021).

27. S. M. Sternson, A.-K. Eiselt, Three pillars for the neural control of appetite. *Annu. Rev. Physiol.* **79**, 401–423 (2017).

28. K. Nagai, H. Ino, H. Yamamoto, H. Nakagawa, M. Yamano, M. Tohyama, S. Shiosaka, Y. Shiotani, S. Inagaki, S. Kitoh, Lesions in the lateral part of the dorsal parabrachial nucleus caused hyperphagia and obesity. *J. Clin. Biochem. Nutr.* **3**, 103–112 (1987).

29. C. Becskei, V. Grabler, G. L. Edwards, T. Riediger, T. A. Lutz, Lesion of the lateral parabrachial nucleus attenuates the anorectic effect of peripheral amylin and CCK. *Brain Res.* **1162**, 76–84 (2007).

30. Z. Zhu, R. Gong, V. Rodriguez, K. T. Quach, X. Chen, S. M. Sternson, Hedonic eating is controlled by dopamine neurons that oppose GLP-1R satiety. *Science* **387**, eadt0773 (2025).

31. B. L. Roth, DREADDs for neuroscientists. *Neuron* **89**, 683–694 (2016).

32. M. E. Carter, M. E. Soden, L. S. Zweifel, R. D. Palmiter, Genetic identification of a neural circuit that suppresses appetite. *Nature* **503**, 111–114 (2013).

33. Y. Qiao, C.-K. Zhang, Z.-H. Li, Z.-H. Niu, J. Li, J.-L. Li, Collateral projections from the lateral parabrachial nucleus to the central amygdaloid nucleus and the ventral tegmental area in the rat. *Anat. Rec.* **302**, 1178–1186 (2019).

34. M. A. Rossi, G. D. Stuber, Overlapping brain circuits for homeostatic and hedonic feeding. *Cell Metab.* **27**, 42–56 (2018).

35. V. Zell, T. Steinkellner, N. G. Hollon, S. M. Warlow, E. Souter, L. Faget, A. C. Hunker, X. Jin, L. S. Zweifel, T. S. Hnasko, VTA glutamate neuron activity drives positive reinforcement absent dopamine co-release. *Neuron* **107**, 864–873.e4 (2020).

36. J. H. Yoo, V. Zell, N. Gutierrez-Reed, J. Wu, R. Ressler, M. A. Shenasa, A. B. Johnson, K. H. Fife, L. Faget, T. S. Hnasko, Ventral tegmental area glutamate neurons co-release GABA and promote positive reinforcement. *Nat. Commun.* **7**, 13697 (2016).

37. E. E. Steinberg, F. Gore, B. D. Heifets, M. D. Taylor, Z. C. Norville, K. T. Beier, C. Földy, T. N. Lerner, L. Luo, K. Deisseroth, R. C. Malenka, Amygdala-midbrain connections modulate appetitive and aversive learning. *Neuron* **106**, 1026–1043.e9 (2020).

38. M. A. Schalla, A. Stengel, Activity based anorexia as an animal model for anorexia nervosa–A systematic review. *Front. Nutr.* **6**, 69 (2019).

39. R. H. S. Nordbø, E. M. S. Espeset, K. S. Gulliksen, F. Skårderud, A. Holte, The meaning of self-starvation: Qualitative study of patients' perception of anorexia nervosa. *Int. J. Eat. Disord.* **39**, 556–564 (2006).

40. J. Patching, J. Lawler, Understanding women's experiences of developing an eating disorder and recovering: A life-history approach. *Nurs. Inq.* **16**, 10–21 (2009).

41. J.-H. Cho, K. Deisseroth, V. Y. Bolshakov, Synaptic encoding of fear extinction in mPFC-amygdala circuits. *Neuron* **80**, 1491–1507 (2013).

42. L. Petreanu, D. Huber, A. Sobczyk, K. Svoboda, Channelrhodopsin-2–assisted circuit mapping of long-range callosal projections. *Nat. Neurosci.* **10**, 663–668 (2007).

43. H. Yang, J. W. de Jong, I. Cerniauskas, J. R. Peck, B. K. Lim, H. Gong, H. L. Fields, S. Lammel, Pain modulates dopamine neurons via a spinal–parabrachial–mesencephalic circuit. *Nat. Neurosci.*, **24**, 1402–1413 (2021).

44. J.-H. Tsou, S.-R. Lee, C.-Y. Chiang, Y.-J. Yang, F.-Y. Guo, S.-Y. Ni, H.-J. Yau, Negative emotions recruit the parabrachial nucleus efferent to the VTA to disengage instrumental food seeking. *J. Neurosci.* **43**, 7276–7293 (2023).

45. S. Fujisawa, G. Buzsáki, A 4 Hz oscillation adaptively synchronizes prefrontal, VTA, and hippocampal activities. *Neuron* **72**, 153–165 (2011).

46. S. Duvarci, E. H. Simpson, G. Schneider, E. R. Kandel, J. Roeper, T. Sigurdsson, Impaired recruitment of dopamine neurons during working memory in mice with striatal D2 receptor overexpression. *Nat. Commun.* **9**, 2822 (2018).

47. M. Lin, D. Sambo, H. Khoshbouei, Methamphetamine regulation of firing activity of dopamine neurons. *J. Neurosci.* **36**, 10376–10391 (2016).

48. R. A. Essner, A. G. Smith, A. A. Jamnik, A. R. Ryba, Z. D. Trutner, M. E. Carter, AgRP neurons can increase food intake during conditions of appetite suppression and inhibit anorexigenic parabrachial neurons. *J. Neurosci.* **37**, 8678–8687 (2017).

49. C. W. Roman, V. A. Derkach, R. D. Palmiter, Genetically and functionally defined NTS to PBN brain circuits mediating anorexia. *Nat. Commun.* **7**, 11905 (2016).

50. M. M. Li, J. C. Madara, J. S. Steger, M. J. Krashes, N. Balthasar, J. N. Campbell, J. M. Resch, N. J. Conley, A. S. Garfield, B. B. Lowell, The paraventricular hypothalamus regulates satiety and prevents obesity via two genetically distinct circuits. *Neuron* **102**, 653–667.e6 (2019).

51. W. Fan, K. L. J. Ellacott, I. G. Halatchev, K. Takahashi, P. Yu, R. D. Cone, Cholecystokinin-mediated suppression of feeding involves the brainstem melanocortin system. *Nat. Neurosci.* **7**, 335–336 (2004).

52. N. C. Barbarich-Marsteller, M. D. Underwood, R. W. Foltin, M. M. Myers, B. T. Walsh, J. S. Barrett, D. A. Marsteller, Identifying novel phenotypes of vulnerability and resistance to activity-based anorexia in adolescent female rats. *Int. J. Eat. Disord.* **46**, 737–746 (2013).

53. C. A. Campos, A. J. Bowen, M. W. Schwartz, R. D. Palmiter, Parabrachial CGRP neurons control meal termination. *Cell Metab.* **23**, 811–820 (2016).

54. C. J. Foldi, L. K. Milton, B. J. Oldfield, A focus on reward in anorexia nervosa through the lens of the activity-based anorexia rodent model. *J. Neuroendocrinol.* **29**, e12479 (2017).

55. S. J. Klenotich, S. C. Dulawa, "The activity-based anorexia mouse model," in *Psychiatric Disorders: Methods and Protocols*, F. H. Kobeissy, Ed. (Humana Press, 2012), pp. 377–393; https://doi.org/10.1007/978-1-61779-458-2_25.

56. J. A. Beeler, D. Mourra, R. M. Zanca, A. Kalmbach, C. Gellman, B. Y. Klein, R. Ravenelle, P. Serrano, H. Moore, S. Rayport, S. Mingote, N. S. Burghardt, Vulnerable and resilient phenotypes in a mouse model of anorexia nervosa. *Biol. Psychiatry* **90**, 829–842 (2021).

57. J. A. Beeler, N. S. Burghardt, The rise and fall of dopamine: A two-stage model of the development and entrenchment of anorexia nervosa. *Front. Psych.* **12**, 799548 (2022).

58. F. Ducrocq, E. Brouwer, K. L. Kooij, I. G. Wolterink-Donselaar, L. Drost, J. Hak, M. Veendijk, M. C. M. Luijendijk, F. J. Meye, R. A. H. Adan, Reduced GABA transmission onto ventral

tegmental area dopamine neurons underlies vulnerability for hyperactivity in a mouse model of Anorexia Nervosa. bioRxiv 585038 [Preprint] (2024). https://doi.org/10.1101/2024.03.14.585038.

59. B. Juarez, M.-S. Kong, Y. S. Jo, J. E. Elum, J. X. Yee, S. Ng-Evans, M. Cline, A. C. Hunker, M. A. Quinlan, M. A. Baird, A. J. Elerding, M. Johnson, D. Ban, A. Mendez, N. L. Goodwin, M. E. Soden, L. S. Zweifel, Temporal scaling of dopamine neuron firing and dopamine release by distinct ion channels shape behavior. *Sci. Adv.* **9**, eadg8869 (2023).

60. M. DeGuzman, M. E. Shott, T. T. Yang, J. Riederer, G. K. W. Frank, Association of elevated reward prediction error response with weight gain in adolescent anorexia nervosa. *Am. J. Psychiatry* **174**, 557–565 (2017).

61. H. J. Watson, Z. Yilmaz, L. M. Thornton, C. Hübel, J. R. I. Coleman, H. A. Gaspar, J. Bryois, A. Hinney, V. M. Leppä, M. Mattheisen, S. E. Medland, S. Ripke, S. Yao, P. Giusti-Rodríguez, K. B. Hanscombe, K. L. Purves, R. A. H. Adan, L. Alfredsson, T. Ando, O. A. Andreassen, J. H. Baker, W. H. Berrettini, I. Boehm, C. Boni, V. B. Perica, K. Buehren, R. Burghardt, M. Cassina, S. Cichon, M. Clementi, R. D. Cone, P. Courtet, S. Crow, J. J. Crowley, U. N. Danner, O. S. P. Davis, M. de Zwaan, G. Dedoussis, D. Degortes, J. E. DeSocio, D. M. Dick, D. Dikeos, C. Dina, M. Dmitrzak-Weglarz, E. Docampo, L. E. Duncan, K. Egberts, S. Ehrlich, G. Escaramís, T. Esko, X. Estivill, A. Farmer, A. Favaro, F. Fernández-Aranda, M. M. Fichter, K. Fischer, M. Föcker, L. Foretova, A. J. Forstner, M. Forzan, C. S. Franklin, S. Gallinger, I. Giegling, J. Giuranna, F. Gonidakis, P. Gorwood, M. G. Mayora, S. Guillaume, Y. Guo, H. Hakonarson, K. Hatzikotoulas, J. Hauser, J. Hebebrand, S. G. Helder, S. Herms, B. Herpertz-Dahlmann, W. Herzog, L. M. Huckins, J. I. Hudson, H. Imgart, H. Inoko, V. Janout, S. Jiménez-Murcia, A. Julià, G. Kalsi, D. Kaminská, J. Kaprio, L. Karhunen, A. Karwautz, M. J. H. Kas, J. L. Kennedy, A. Keski-Rahkonen, K. Kiezebrink, Y.-R. Kim, L. Klareskog, K. L. Klump, G. P. S. Knudsen, M. C. La Via, S. Le Hellard, R. D. Levitan, D. Li, L. Lilenfeld, B. D. Lin, J. Lissowska, J. Luykx, P. J. Magistretti, M. Maj, K. Mannik, S. Marsal, C. R. Marshall, M. Mattingsdal, S. McDevitt, P. McGuffin, A. Metspalu, I. Meulenbelt, N. Micali, K. Mitchell, A. M. Monteleone, P. Monteleone, M. A. Munn-Chernoff, B. Nacmias, M. Navratilova, I. Ntalla, J. K. O'Toole, R. A. Ophoff, L. Padyukov, A. Palotie, J. Pantel, H. Papezova, D. Pinto, R. Rabionet, A. Raevuori, N.

Ramoz, T. Reichborn-Kjennerud, V. Ricca, S. Ripatti, F. Ritschel, M. Roberts, A. Rotondo, D. Rujescu, F. Rybakowski, P. Santonastaso, A. Scherag, S. W. Scherer, U. Schmidt, N. J. Schork, A. Schosser, J. Seitz, L. Slachtova, P. E. Slagboom, M. C. T. Slof-Op 't Landt, A. Slopien, S. Sorbi, B. Świątkowska, J. P. Szatkiewicz, I. Tachmazidou, E. Tenconi, A. Tortorella, F. Tozzi, J. Treasure, A. Tsitsika, M. Tyszkiewicz-Nwafor, K. Tziouvas, A. A. van Elburg, E. F. van Furth, G. Wagner, E. Walton, E. Widen, E. Zeggini, S. Zerwas, S. Zipfel, A. W. Bergen, J. M. Boden, H. Brandt, S. Crawford, K. A. Halmi, L. J. Horwood, C. Johnson, A. S. Kaplan, W. H. Kaye, J. E. Mitchell, C. M. Olsen, J. F. Pearson, N. L. Pedersen, M. Strober, T. Werge, D. C. Whiteman, D. B. Woodside, G. D. Stuber, S. Gordon, J. Grove, A. K. Henders, A. Juréus, K. M. Kirk, J. T. Larsen, R. Parker, L. Petersen, J. Jordan, M. Kennedy, G. W. Montgomery, T. D. Wade, A. Birgegård, P. Lichtenstein, C. Norring, M. Landén, N. G. Martin, P. B. Mortensen, P. F. Sullivan, G. Breen, C. M. Bulik, Genome-wide association study identifies eight risk loci and implicates metabo-psychiatric origins for anorexia nervosa. *Nat. Genet.* **51**, 1207–1214 (2019).

62. V. Boraska, C. S. Franklin, J. A. B. Floyd, L. M. Thornton, L. M. Huckins, L. Southam, N. W. Rayner, I. Tachmazidou, K. L. Klump, J. Treasure, C. M. Lewis, U. Schmidt, F. Tozzi, K. Kiezebrink, J. Hebebrand, P. Gorwood, R. A. H. Adan, M. J. H. Kas, A. Favaro, P. Santonastaso, F. Fernández-Aranda, M. Gratacos, F. Rybakowski, M. Dmitrzak-Weglarz, J. Kaprio, A. Keski-Rahkonen, A. Raevuori, E. F. Van Furth, M. C. T. Slof-Op 't Landt, J. I. Hudson, T. Reichborn-Kjennerud, G. P. S. Knudsen, P. Monteleone, A. S. Kaplan, A. Karwautz, H. Hakonarson, W. H. Berrettini, Y. Guo, D. Li, N. J. Schork, G. Komaki, T. Ando, H. Inoko, T. Esko, K. Fischer, K. Männik, A. Metspalu, J. H. Baker, R. D. Cone, J. Dackor, J. E. DeSocio, C. E. Hilliard, J. K. O'Toole, J. Pantel, J. P. Szatkiewicz, C. Taico, S. Zerwas, S. E. Trace, O. S. P. Davis, S. Helder, K. Bühren, R. Burghardt, M. de Zwaan, K. Egberts, S. Ehrlich, B. Herpertz-Dahlmann, W. Herzog, H. Imgart, A. Scherag, S. Scherag, S. Zipfel, C. Boni, N. Ramoz, A. Versini, M. K. Brandys, U. N. Danner, C. de Kovel, J. Hendriks, B. P. C. Koeleman, R. A. Ophoff, E. Strengman, A. A. van Elburg, A. Bruson, M. Clementi, D. Degortes, M. Forzan, E. Tenconi, E. Docampo, G. Escaramís, S. Jiménez-Murcia, J. Lissowska, A. Rajewski, N. Szeszenia-Dabrowska, A. Slopien, J. Hauser, L. Karhunen, I. Meulenbelt, P. E. Slagboom, A. Tortorella, M. Maj, G. Dedoussis, D. Dikeos, F. Gonidakis, K. Tziouvas, A. Tsitsika, H. Papezova, L. Slachtova, D. Martaskova, J. L. Kennedy, R. D. Levitan, Z. Yilmaz, J. Huemer, D. Koubek, E.

Merl, G. Wagner, P. Lichtenstein, G. Breen, S. Cohen-Woods, A. Farmer, P. McGuffin, S. Cichon, I. Giegling, S. Herms, D. Rujescu, S. Schreiber, H.-E. Wichmann, C. Dina, R. Sladek, G. Gambaro, N. Soranzo, A. Julia, S. Marsal, R. Rabionet, V. Gaborieau, D. M. Dick, A. Palotie, S. Ripatti, E. Widén, O. A. Andreassen, T. Espeseth, A. Lundervold, I. Reinvang, V. M. Steen, S. Le Hellard, M. Mattingsdal, I. Ntalla, V. Bencko, L. Foretova, V. Janout, M. Navratilova, S. Gallinger, D. Pinto, S. W. Scherer, H. Aschauer, L. Carlberg, A. Schosser, L. Alfredsson, B. Ding, L. Klareskog, L. Padyukov, P. Courtet, S. Guillaume, I. Jaussent, C. Finan, G. Kalsi, M. Roberts, D. W. Logan, L. Peltonen, G. R. S. Ritchie, J. C. Barrett, X. Estivill, A. Hinney, P. F. Sullivan, D. A. Collier, E. Zeggini, C. M. Bulik, A genome-wide association study of anorexia nervosa. *Mol. Psychiatry* **19**, 1085–1094 (2014).

63. B. Peng, Q. Xu, J. Liu, S. Guo, S. L. Borgland, S. Liu, Corticosterone attenuates reward-seeking behavior and increases anxiety via D2 receptor signaling in ventral tegmental area dopamine neurons. *J. Neurosci.* **41**, 1566–1581 (2021).

64. D. P. Nguyen, S. P. Layton, G. Hale, S. N. Gomperts, T. J. Davidson, F. Kloosterman, M. A. Wilson, Micro-drive array for chronic in vivo recording: Tetrode assembly. *J. Vis. Exp.*, e1098 (2009).

65. M. Schroeder, M. Jakovcevski, T. Polacheck, Y. Drori, A. Luoni, S. Röh, J. Zaugg, S. Ben-Dor, C. Albrecht, A. Chen, Placental miR-340 mediates vulnerability to activity based anorexia in mice. *Nat. Commun.* **9**, 1596 (2018).

66. L. Boekhoudt, A. Omrani, M. C. M. Luijendijk, I. G. Wolterink-Donselaar, E. C. Wijbrans, G. van der Plasse, R. A. H. Adan, Chemogenetic activation of dopamine neurons in the ventral tegmental area, but not substantia nigra, induces hyperactivity in rats. *Eur. Neuropsychopharmacol.* **26**, 1784–1793 (2016).

67. Y. Humeau, F. Doussau, N. J. Grant, B. Poulain, How botulinum and tetanus neurotoxins block neurotransmitter release. *Biochimie* **82**, 427–446 (2000).

68. J. N. Gelinas, D. Khodagholy, Interictal network dysfunction and cognitive impairment in epilepsy. *Nat. Rev. Neurosci.* **26**, 399–414 (2025).

69. J. H. Jennings, D. R. Sparta, A. M. Stamatakis, R. L. Ung, K. E. Pleil, T. L. Kash, G. D. Stuber, Distinct extended amygdala circuits for divergent motivational states. *Nature* **496**, 224–228 (2013).

70. T. Kimm, Z. M. Khaliq, B. P. Bean, Differential regulation of action potential shape and burst-frequency firing by BK and Kv2 channels in substantia nigra dopaminergic neurons. *J. Neurosci.* **35**, 16404–16417 (2015).

71. M. Sekerli, C. A. Del Negro, R. H. Lee, R. J. Butera, Estimating action potential thresholds from neuronal time-series: New metrics and evaluation of methodologies. *IEEE Trans. Biomed. Eng.* **51**, 1665–1672 (2004).

72. J. Platkiewicz, R. Brette, A threshold equation for action potential initiation. *PLOS Comput. Biol.* **6**, e1000850 (2010).
